# Heteroleptic Coumarin-Based Silver(I) Complexes: Possible New Antimicrobial Agents

**DOI:** 10.3390/molecules29245917

**Published:** 2024-12-15

**Authors:** Erika Mooney, Brendan Twamley, Gordon Cooke, Emma Caraher, Matthias Tacke, Fintan Kelleher, Bernadette S. Creaven

**Affiliations:** 1 School of Chemical and BioPharmaceutical Sciences, Technological University Dublin, TU Dublin, Tallaght Campus, D24 FKT9 Dublin, Ireland; x00117667@mytudublin.ie (E.M.); gordon.cooke@tudublin.ie (G.C.);; 2 Centre for AMR and One Health Research, Technological University Dublin, TU Dublin, Tallaght Campus, D24 FKT9 Dublin, Ireland; 3 School of Chemistry, Trinity College Dublin, D02 PN40 Dublin, Ireland; 4 UCD School of Chemistry, Science Centre South, University College Dublin, Belfield, D04 V1W8 Dublin, Ireland; matthias.tacke@ucd.ie; 5 School of Chemical and BioPharmaceutical Sciences, Technological University Dublin, Central Quad Building, Grangegorman, D07 ADY7 Dublin, Ireland

**Keywords:** silver(I), coumarin, hybrid, antimicrobial, photostability

## Abstract

Heteroleptic coumarin-based silver(I) complexes with improved solubility profiles were synthesised using either triphenylphosphine or an *N*-heterocyclic carbene as adduct ligands, and were fully characterised using IR and NMR spectroscopy, elemental analysis, and, where possible, X-ray crystallography. The triphenylphosphine adducts formed well-resolved structures, where the oxyacetate ligands asymmetrically chelated the silver(I) ion in a bidentate chelating mode, and the silver(I) ion was also bound to two triphenylphosphine ligands. The solubility profile and photostability of the adducts were considerably improved compared to those of previously isolated simple coumarin silver(I) complexes. Analysis of the coumarin *N*-heterocyclic carbene(NHC) silver(I) adduct indicated that it likely formed as a complex aggregate species with an overall stoichiometry of 1:1:1 coumarin:Ag(I):NHC. The Kirby Bauer assay and broth microdilution assays were used to assess the silver(I) complexes’ and adducts’ antimicrobial activity against pathogenic strains of *Pseudomonas aeruginosa*, *Escherichia coli*, and MRSA. Interestingly, the formation of more soluble complexes did not increase the activity of the silver(I) complexes and, in effect, made them less effective antimicrobial agents, particularly against *Escherichia coli* and *Pseudomonas aeruginosa*, although they retained their activity against MRSA.

## 1. Introduction

The alarming speed at which bacteria evolve, acquire, and develop novel mechanisms of resistance to evade the drug-mediated killing associated with currently used antibiotic drug therapies is leading to a global health threat, with the potential to become the next pandemic [1]. Today, the problem posed by Antimicrobial Resistance (AMR) is considered one of the top ten global health threats, with approximately 13·7 million infection-related deaths reported in 2019 [2]. Moreover, by 2050, this problem is projected to cause more than 10 million global deaths annually [3]. The recent COVID-19 pandemic has exacerbated this worldwide health problem, as critical antibiotics have been overused in the treatment of secondary infections in patients associated with severe COVID-19 illness [4].

In 2022, a Global Burden of Disease study identified *Escherichia coli*, *Staphylococcus aureus*, *Klebsiella pneumoniae*, *Streptococcus pneumoniae*, *Acinetobacter baumannii*, and *Pseudomonas aeruginosa* as the six leading causes (73%) of AMR-associated deaths in 2019 [3]. Previous studies from our group, which investigated the antimicrobial activity of metal-based coumarin complexes against *Escherichia coli* (*E. coli*), *Pseudomonas aeruginosa* (*P. aeruginosa*) and *Staphylococcus aureus* (*S. aureus*), showed that silver(I) complexes of coumarin-derived ligands were particularly active against these pathogens [5,6,7,8]. The effectiveness of any silver drug as an antibacterial agent is linked to its bioavailability, and while silver(I) compounds have shown excellent antibiotic activity, their therapeutic use has been limited to date, primarily as a topical agent. Silver nitrate and silver sulfadiazine, commonly used topical antibacterials, have a very rapid bactericidal action, but they lose their efficacy as the complexes are ionic in nature, leading to a labile silver(I) ion being formed [9]. Quick release is optimal to kill surrounding bacteria, but the wound site remains exposed to possible re-infection once the initial burst of silver(I) ions dissipates.

The previously isolated series of coumarin-based silver(I) complexes, while effective as antibacterial agents, are largely polymeric in nature, and their solubility in aqueous solutions and photostability are limited. The choice of using coumarin-derived ligands is because coumarin is a privileged scaffold, and its many derivatives occur widely in nature. Many coumarin derivatives, both plant-derived and synthetic, have been found to have a range of therapeutic properties [10]; however, metal complexes of coumarin derivatives are often polymeric in nature and have poor solubility [11]. A 2023 review of recent advances in the use of coumarin-hybrid complexes has identified a number of such compounds which are currently in clinical trials for a range of diseases and medical conditions [12]. The approach of using complexes with multiple pharmacophores to address the challenges posed by our previous studies, namely their lack of solubility in aqueous media, led to our most recent work, where we reported the synthesis and characterisation of *N*-heterocyclic carbene (NHC) adducts of silver(I) coumarin carboxylate complexes [13]. *N*-heterocyclic carbenes (NHC), due to their excellent σ-donating properties and π-back bonding ability, are widely used as ancillary ligands to stabilise both the main group and transition metals [14,15], while the addition of triphenylphosphine (TPP) ligands has been shown to improve the photostability of silver(I) carboxylate complexes [16]. The introduction of a second ligand to the coumarin carboxylate complexes improved the solubility and photostability of the resulting adducts, and the formation of heteroleptic triphenylphosphino and NHC adducts improved their antimicrobial activity against some strains [13]. The synthesis of bis-triphenylphosphino-derived adducts proved to be straightforward. However, the synthesis and isolation of 1:1:1 coumarin:silver:NHC adducts proved difficult with very low yields, and the product of the reaction between the NHC and the silver coumarin carboxylates under Schlenk conditions resulted in a heteroleptic complex with two silver ions forming a strong argentophilic Ag(I)−Ag(I) interaction, with one silver(I) ion bound to two coumarin carboxylate ligands and the second ion bound to two carbene ligands in a Schlenk-like equilibrium complex. Isolation via an ionic carbene route led to higher yields of adducts, but more complex aggregates were formed. MS studies indicated that the complexes dissociated in polar solvents into ion-pair aggregates; therefore, an alternative strategy was proposed in this work to allow for the successful isolation of 1:1:1 coumarin: silver:NHC adducts which would have improved bioavailability, solubility, and photostability profiles.

**Figure 1:**
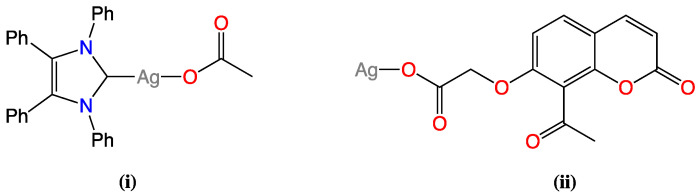
Chemical structures of silver(I) complexes (**i**) SBC3 and (**ii**) 2-(8-acetyl-2-oxo-2H-chromen-7-yl)oxyacetatesilver(I).

The approach used in this study was to use the complex, 2-(8-acetyl-2-oxo-2H-chromen-7-yl)oxyacetato silver(I), as shown in Figure 1(ii), which has been previously synthesised and reported by our group to have credible antimicrobial activity against the clinically important MRSA and P. aeruginosa with MIC_50_ values of 33.0 and 22.7 µM respectively [7]. The acetate group at position C^7^ of this complex is comparable to that of the acetate function present in the antimicrobial compound known as SBC3, shown in Figure 1(i), synthesised using the ionic route by O’Beirne et al. [17]. SBC3 has excellent antimicrobial activity against bacterial strains of P. aeruginosa and *E. coli*, with MIC values of 3.13 and 6.25 μg/mL, respectively, while its activity against MRSA showed an MIC value of 12.5 μg/mL [17]. This compound also has high solubility in aqueous media and good stability in light. Thus, using 2-(8-acetyl-2-oxo-2H-chromen-7-yl)oxyacetatosilver(I) instead of the silver acetate moiety in SBC3 was proposed as a strategy to increase antimicrobial activity, with the possibility of dual-mode activity arising from the presence of both carbene and coumarin moieties, in addition to improving the physicochemical and bioavailability characteristics of the resulting adducts. The pKa values of the carboxylate group will not be greatly different for the oxyacetate moiety relative to the coumarin-3-carboxylate moiety used in previous studies, but there would be less steric hindrance around the silver(I) ion from the oxyacetate binding group.

To address the challenges associated with the previously poor solubility of promising lead compounds, we synthesised bis-TPP adducts for a previous series of coumarin silver(I) complexes [13], and their resulting improved physicochemical properties also prompted the synthesis of bis-TPP adducts in this study. We have also extended the biological studies to look at the activity of coumarin-derived silver(I) complexes against reference laboratory strains of *E. coli*, *P. aeruginosa* and MRSA.

## 2. Results and Discussion

### 2.1. Synthesis and Spectroscopic Characterisation of 2-(2-Oxo-2H-chromen-7-yl)-oxyacetatobistriphenylphosphinosilver(I) Complexes

**Scheme 1:**
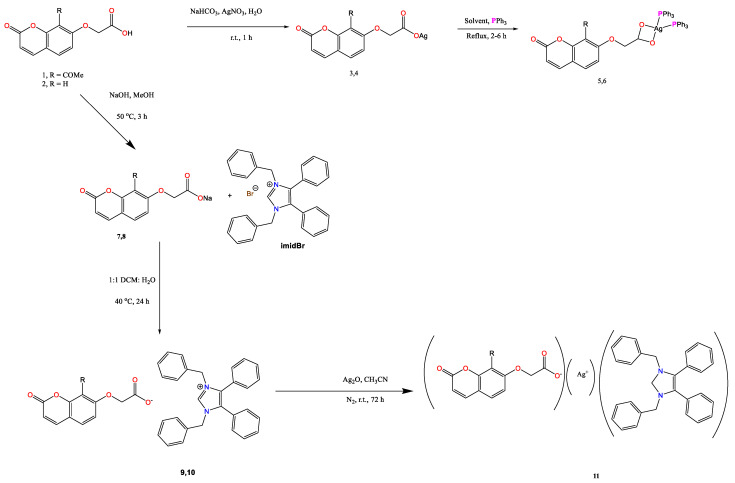
Synthetic protocols used toward the synthesis of bis-triphenylphosphino adducts (**5** and **6**) and substituted coumarin-7-oxyaceto-silver-NHC* complexes (**11**) R = -COCH_3_ for **5**, **7**, **9**, and **11** and R = H for **6**, **8**, and **10**.

The silver(I) complexes of coumarin-7-oxyacetate ligands **3** and **4**, shown in Scheme 1, were prepared by previously published methods from ligands **1** and **2**, with details given in the Supplementary Information. Adducts **5** and **6** were obtained by reacting complexes **3** and **4** in a 1:2 molar ratio with triphenylphosphine, using either ethanol or dichloromethane (DCM) as the reaction solvent. Both adducts were isolated as white-coloured powders in yields of 71 and 78%, respectively. Recrystallisation of **5** from DCM/ethanol afforded colourless crystals suitable for X-ray crystallographic analysis, while crystals of **6** were obtained through slow diffusion of pentane into a saturated solution of **6** in DCM.

The bis-triphenylphosphino adducts **5** and **6** were analysed by IR, ^1^H and ^13^C NMR spectroscopy, and elemental analysis. The main IR bands of the TPP adducts are presented in Table 1, showing a shift in the position of the IR bands for the carbonyl stretch of the lactone functionality, as well as the asymmetric and symmetric stretches of the carboxylate group relative to the silver(I) complexes **3** and **4** (Table S4.2—Supplementary Data). The Δν_(oco)_ values for the silver(I) TPP adducts were in the range of ca. 125–150 cm^−1^ and are typical for bidentate chelating bonding [13,17]. In addition, bands typical of those found in TPP (P-C band: 795–650 cm^−1^ and P-Ph: 1130–1090 cm^−1^) [17] were present in the IR spectra of each of the TPP adducts, further confirming their formation. Microanalytical data confirmed the inclusion of a molecule of DCM for complex **6**.

**Figure 2:**
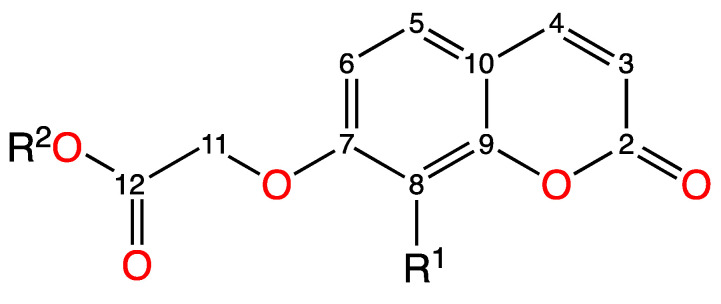
General numbering system for the assignment of NMR spectra of **3**–**6**.

The NMR spectral data for adducts **5** and **6** are presented in Supplementary Information together with the data for silver(I) complexes **3** and **4** and ligands (Figure S3.1a,b and Table S4.3). A summary of the data is given in Table 2. An upfield movement of the signals for protons H^5^ and H^11^ (Figure 2) of adducts **5** and **6** was observed compared to the corresponding signals for protons of the silver(I) complexes **3** and **4**. In particular, the signal for H^11^, which is closest to the site of adduct formation, experienced the greatest upfield chemical movement for both adducts (Table 2). The increased electron density around the central silver(I) ion provided by the two TPP ligands is likely responsible for this chemical shift movement in the signals for the coumarin ligand. For both adducts, new multiple proton signals were observed in the region 7.53–7.30 ppm, with relative integration of 30 protons, confirming the presence of two TPP ligands. The coumarin ^13^C NMR signals were less affected by adduct formation, with the formation of **5** and **6** confirmed mainly by the addition of four new carbon signals to the ^13^C NMR spectra, corresponding to the aromatic carbons of the triphenylphosphine ligand (Table S4.4 in supplemental data).

The poor solubility of the parent silver(I) complexes **3** and **4** in common laboratory solvents other than dimethyl sulfoxide (DMSO) has been a limiting factor toward the potential development of these silver(I) complexes as therapeutic agents. The *bis*-TPP adducts **5** and **6** were readily soluble in DMSO at room temperature, and the solubility of the complexes also extended to toluene, chloroform, DCM, tetrahydrofuran and acetonitrile at room temperature. While heating to ~50 °C extends their solubility further to more polar solvents such as ethyl acetate, methanol, and ethanol. Both adducts remained stable in solution over 96 h in the absence of UV-Vis light (Figure S3.2a–d). However, upon exposure to ambient light conditions, degradation of **five** was observed to begin after 24 h, while complex **6** remained stable.

### 2.2. X-Ray Crystallography of Adducts ***5*** and ***6***

**Figure 3:**
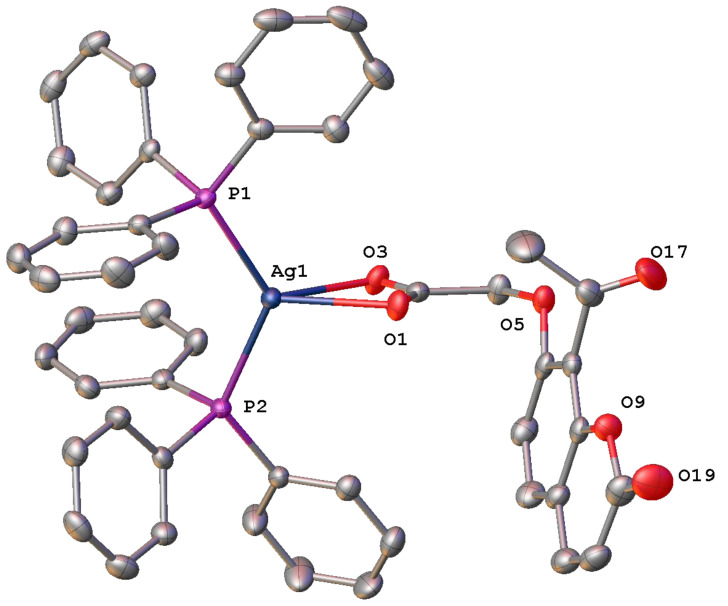
Molecular structure of [bis-triphenylphosphino)-(8-acetyl-2-oxo-2H-chromen-7-oxyacetosilver(I)] (**5**) with displacement at 50% probability, hydrogen atoms and disordered solvent omitted for clarity. Heteroatoms labelled only. See Figure S3.3a.

**Figure 4:**
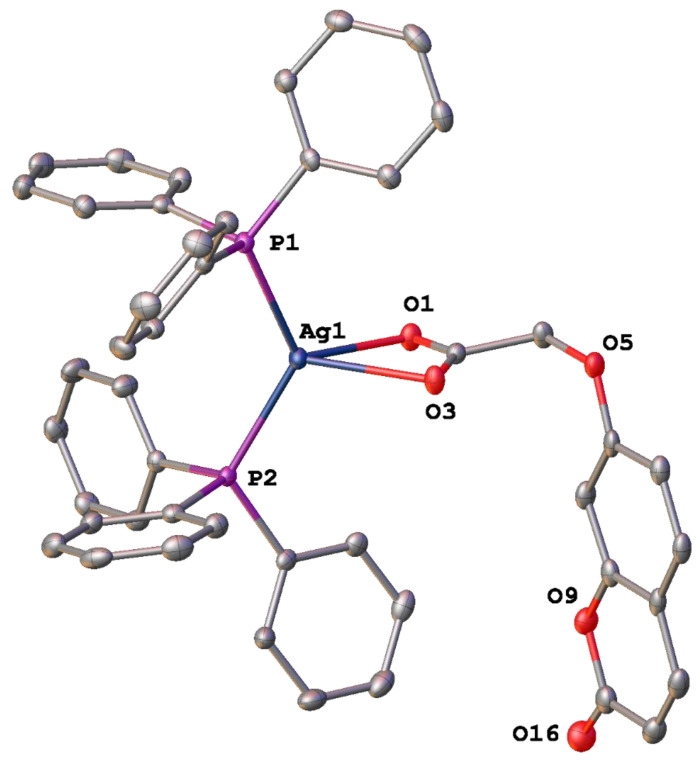
Molecular structure of [bis-triphenylphosphino)-(2-oxo-2H-chromen-7-oxyacetosilver(I)] (**6**) with displacement at 50% probability, hydrogen atoms and solvent omitted for clarity. Heteroatoms labelled only. See Figure S3.4a.

For each of the bis-triphenylphosphino adducts synthesised, crystals suitable for X-ray analysis were obtained. In the case of **5**, clear colourless block-like crystals were obtained through recrystallisation from DCM/ethanol, while slow diffusion of pentane into a saturated solution of **6** in DCM at 4 °C afforded colourless rod-shaped crystals. Presented in Table 3 are the selected bond lengths and angles of each adduct, while the crystal data and structure refinement details of the two TPP adducts **5** and **6** are provided in Table S4.5 in the Supplementary Information. The X-ray crystal structures depicted in Figure 3 and Figure 4 show that the oxyacetate ligands asymmetrically chelate the silver(I) ion in a bidentate chelating mode in both complexes. Similar to the [bis-(triphenylphosphino)-(2-oxo-2H-chromen-3-carboxylato)]silver(I) complexes isolated previously [13], this binding mode is also consistent with the IR spectral data as both complexes produced a Δν_(oco)_ of <150 cm^−1^. Both complexes grew as solvate forms with a disordered mixed solvent of DCM/ethanol with an occupancy of 25% per molecule of **5**, while **6** had a single DCM molecule in the structure (Figures S3.3a and S3.4a)

The Ag-O bond lengths in these adducts, **5** (2.383 (2) and 2.538 (2) Å), **6** (2.4548 (16) and 2.4061 (16) Å) are noticeably longer than those expected for bond lengths such as these (2.2–2.4 Å) [18] but are comparable to other observed by other adducts of this type such as for the [bis-(triphenylphosphino)-(2-oxo-2H-chromen-3-carboxylato)]silver(I) complexes [13]. They are also comparable to the bis-triphenylphosphino silver(I) adduct reported by Edwards et al. [18], where the Ag-O bond lengths were reported as 2.495 (5) and 2.532 (5) Å.

In contrast, the Ag-P bond lengths of each of the adducts formed, **5** (2.4060 (8) and 2.4317 (8) Å) and **6** (2.4104 (6) and 2.4040 (6) Å), like the [bis-(triphenylphosphino)-(2-oxo-2H-chromen-3-carboxylato)]silver(I) complexes, are within the known range for triphenylphosphine bound silver, which is 2.352–2.521 Å [13,18,19].

Both adducts possess a similar P(2)-Ag-P(1) bond angle of 127.53(3)° and 126.62(2)° respectively, much larger than that typical bond angle of 109.5° displayed by a complex with a tetrahedral geometry [20]. They have adopted a distorted tetrahedral geometry likely caused by the crowded environment created by the six phenyl rings around the silver atom. The hydrogen bond geometries are presented in Table 4, and hydrogen bonding is shown in Figures S3.3b and S3.4b.

### 2.3. Synthesis of [(1,3-Dibenzyl-4,5-diphenyl imidazole-2-ylidene)–(8-acetyl-2-oxo-2H-chromene-7-yl)oxyaceto)(silver(I))] (***11***) via an Ion Exchange Route

Synthesis of the *N*-heterocyclic carbene (NHC), as shown in Scheme 1, was via the ionic route previously used by our group, as yields from these reactions are typically high [13]. To synthesise intermediate compounds **7** and **8**, the sodium salts of the carboxylate ligands were first obtained by stirring the respective coumarin acetoxy acid with sodium hydroxide in a 1:1 molar ratio at 50 °C in methanol, forming **7** and **8** in yields of 83% and 91% respectively. Formation of the sodium salts was confirmed by IR spectroscopy and microanalysis as poor solubility did not allow for measurement of ^1^H and ^13^C NMR spectra. A summary of the main IR bands and microanalytical data is given in Table 5. A shift in the position of the carbonyl stretch of the lactone functionality compared to the corresponding acid, coupled with the loss of the broadband corresponding to the -OH stretch of the carboxylic acid in the region 3466–3046 cm^−1^, indicates the successful isolation of the sodium salts. The difference between the asymmetric and symmetric stretches of the carboxylate group of each of the compounds is greater than 200 cm^−1^, which indicates that the binding mode of these compounds is likely to be unidentate or ionic. The microanalytical data indicated that both contained one molecule of water.

The sodium salts **7** and **8** were reacted with imidBr, using a 1.5:1 mmol ratio to form ionic intermediates **9** and **10** in yields of 90 and 96%, respectively, with formation confirmed by ^1^H and ^13^C NMR spectroscopy and IR spectroscopy, while microanalytical data was obtained only for compound **9** as compound **10** was isolated repeatedly as an oil. Table 5 details the main IR bands observed for **9** and **10**, including the bands corresponding to the C-N and C-C stretches of the imidazole ring of the NHC ligand at ~1555 and 1455 cm^−1^, respectively. Coupled with a slight shift in the IR bands corresponding to the ν_CO_ of the lactone function and the ν_asym_ and ν_sym_ stretches of the carboxylate group of the oxyacetate function, these observations indicated the formation of compounds **9** and **10**.

**Figure 5:**
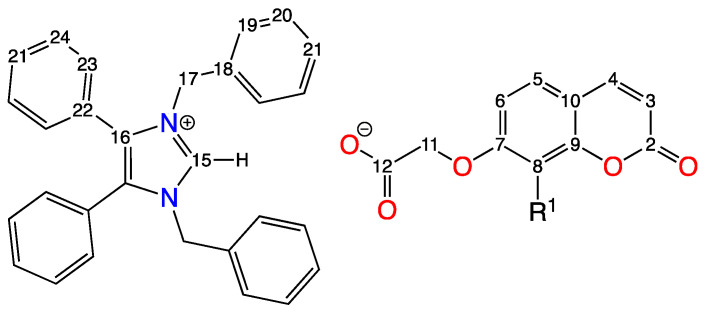
Numbering system used for the assignment of the ^1^H and ^13^C NMR spectra of compounds **9** and **10**, where R^1^ = -COCH_3_ for **9** (labelled C^13^ and C^14^) and = H for **10**.

The main details of the ^1^H and ^13^C NMR spectra of complexes **9** and **10** are given in Table 6, with the numbering system used for the assignment of the ^1^H and ^13^C NMR signals of these NHC^+^ intermediates shown in Figure 5 and the full data provided in Table S4.6 in the Supplementary Information. An example of a typical ^1^H NMR spectrum observed for these intermediate compounds is shown in Figure S3.5.

^1^H NMR signals corresponding to the coumarin moieties experienced an upfield movement, while those signals associated with the NHC^+^ were moved downfield compared to the respective precursors (Supplementary Information Table S4.6). In addition to this, the singlet at ~9.72 ppm, corresponding to the proton attached to the carbenic carbon of the NHC^+^ ligand (H^15^), for both compounds is present. Evidence that compounds **9** and **10** exist as ion pairs is provided by the lack of a signal in both ^1^H NMR spectra, corresponding to the proton of the coumarin carboxylic acid. When the ^1^H NMR spectra of **9** and **10** were compared to those of the corresponding acids **1** and **2**, the signals for the H^11^ protons of both compounds, situated closest to the site of the ionic interaction between the coumarin oxyacetate and NHC^+^ moieties, moved upfield from 4.91 to 4.37 ppm and 4.83 to 4.38 ppm, respectively.

Signals corresponding to both coumarin and NHC^+^ moieties were present in both ^13^C NMR spectra, with full assignments provided in Supplementary Data, Table S4.6. Evidence of the ionic interaction between the ligands of compounds **9** and **10** is also provided by the presence of a C-H signal at 136.7 ppm and 137.3 ppm associated with the carbenic carbon (C^15^) of the NHC^+^ moiety for **9** and **10**, respectively. Similarly, a downfield movement in the signal for the quaternary carbon associated with the carbonyl carbon of the carboxylate group of the coumarin (C^11^) close to the site of the ligand interaction, from 65.1 to 68.3 ppm and 64.8 to 68.6 ppm for **9** and **10**, respectively, was observed, while a slight upfield movement in the C^12^ signal was also observed.

**Figure 6:**
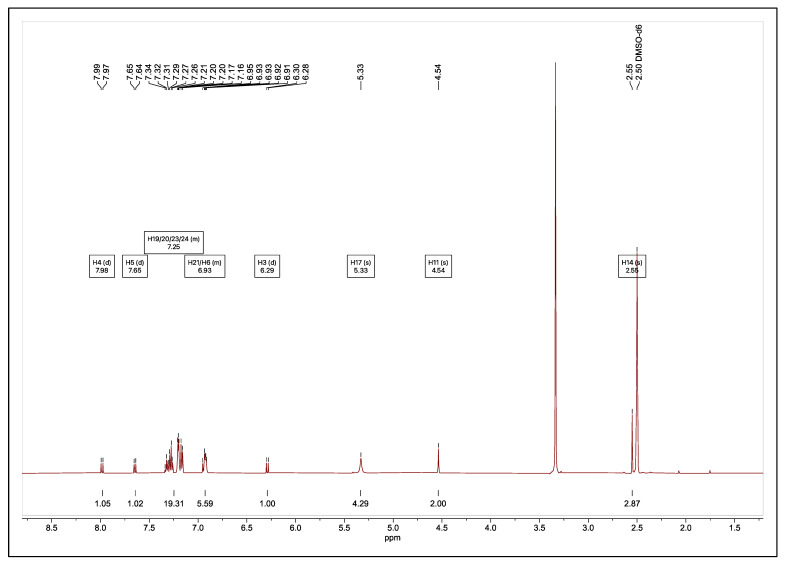
^1^H NMR spectrum of [(8-acetyl-C-7-oxyaceto)(Ag)(NHC)] (**11**) recorded in DMSO-d_6_.

To synthesise the silver(I) complexes of **9** and **10**, the ionic intermediate compounds were stirred in anhydrous acetonitrile containing a 0.2 mmol excess of silver oxide for 72 h at room temperature in a nitrogen-charged flask with the exclusion of light to isolate complex **11** in 82% yield. Despite repeated attempts, the silver(I) complex of **10** could not be isolated in its pure form. Adduct **11** was isolated as an amorphous solid; therefore, its formation was first confirmed by NMR spectroscopy. The ^1^H NMR spectrum of **11** is provided in Figure 6 with the same numbering system (Figure 5) used for the assignment of the ^1^H and ^13^C NMR spectra of **9.** The H^11^ NMR signal of **11** moved downfield from 4.37 to 4.54 ppm, while all other chemical shifts of the protons associated with the coumarin ligand remained largely the same. In contrast, the NMR signals for the protons associated with the NHC* ligand of **11** moved upfield when compared to those of the ionic intermediate **9**. In particular, the NMR signals for the protons of the two -CH_2_ groups (H^17^), closest to the silver ion, experienced the greatest shielding effect moving upfield from 5.42 to 5.33 ppm. The ^13^C NMR signals of the coumarin ligand experienced a slight upfield shift upon the formation of the carbene adduct complex, similar to the carbon signals of the NHC* ligand.

When the main IR bands of **11** (Table 5) were compared to those of the corresponding ionic intermediate, the appearance of a new band at 1398 cm^−1^, corresponding to an asymmetric stretch of this carboxylate group, indicated the formation of the complex. The difference in the symmetric and asymmetric stretches of the carboxylate function is slightly >200 cm^−1^, which would typically indicate an unidentate coordination mode of the coumarin ligand. Previous coumarin−carbene heteroleptic complexes isolated via the in situ generation of a free carbene under Schlenk conditions, followed by reaction with a coumarin silver(I) complex, resulted in the formation of a complex with two silver ions, forming a strong argentophilic Ag(I)−Ag(I) interaction with one silver(I) ion bound via unidentate coordination to two coumarin carboxylate ligands, and the other silver ion bound to two carbene ligands. Isolation via the ionic carbene route used here led to more complex aggregates, and the IR spectra of the amorphous solids indicated that monodentate or ionic bonding of the carboxylate to the silver ions is likely in all cases, given the values of Δν_(oco)_ of >200 cm^−1^ recorded. However, the broad IR bands observed for the asymmetric and symmetric carboxylate bands do not preclude different coordination modes for adduct complexes in the solid state. Microanalytical data indicate that the complex formed is consistent with an empirical formulation of a 1:1:1 ratio of coumarin:Ag:NHC. Extensive efforts were made to form crystals of the complex, but these were not successful; therefore, MS analysis was performed to determine the exact structure of the complex. The MS data for **11** show the presence of a [NHC-Ag-NHC]^+^ moiety (Table S4.7); thus, as in the case of the previous coumarin−carbene silver(I) adducts isolated by ion exchange, the complexes may exist essentially as ion pairs held together by strong argentophilic interactions. However, such species may well form in the gas phase; therefore, the actual structure of the adduct complex remains unresolved. There was no evidence for the [coumarin-Ag-coumarin]^−^ ion, as it appeared to be unstable in the MS experiments. However, in each case, the parent coumarin ligand was observed. The observed isotopic pattern for the [NHC-Ag-NHC]^+^ species of **11** was identical to that reported by Khe et al. for dinuclear silver(I)-NHC complexes [21]. In addition, it has been suggested by other workers that polar solvents such as methanol have been shown to stabilise the ionic [NHC-Ag-NHC]^+^ species and may possibly shift the equilibrium towards bis NHC-Ag complexes [22,23]. The photostability of **11** was examined by ^1^H NMR spectroscopy in deuterated DMSO and CDCl_3_ over a 96 h time period in the absence and presence of light and displayed excellent light stability in DMSO under both conditions (Figure S3.6a–d). Compared to the parent silver(I) complex **3**, which was only soluble in hot DMSO, the solubility of **11** extended to multiple different solvents such as toluene, ethyl acetate, ethanol, methanol, dichloromethane and acetonitrile.

Isolation of the full suite of 7-acetoxy coumarin adduct complexes was not successful; thus, the Kirby Bauer method was used to assess the antibacterial activity of the 8-acetoxy-derived silver(I) complex **3** and its corresponding triphenylphosphino-derived adduct **5** and NHC carbene-derived adduct (**11**), and the results are given below. For comparison, we included coumarin-3-carboxylatosilver(I) [Coumarin-3-COOAg(I)] (**12**), a compound previously isolated by us which had shown excellent antimicrobial activity against MRSA and *E. coli* [24]. In addition, commonly prescribed antibiotics were included in these studies for reference.

#### 2.3.1. Determination of Growth Inhibitory Activity of 8-Acetyl-C-7-oxyacetoAg(I)] and Its Triphenylphosphine and NHC Adducts Against MRSA and *E. coli*

The antimicrobial susceptibility of *E. coli* (ATCC-25922) and MRSA (ATCC-4330) to ligand **1**, silver(I) complex **3**, coumarin-3-COOAg(I) (**12**), and the bis-triphenylphosphino and NHC adducts **5** and **11**, respectively, were determined via the Kirby Bauer disk diffusion assay [25]. Ligand **1** was found to be inactive against both *E. coli* and MRSA, with the diameters of the zones of inhibition measuring 0 mm. In addition, commercially available coumarin-3-carboxylic acid and triphenylphosphine were also found to have no inhibitory activity on the growth of the bacterial strains tested.

The data presented in Table 7 show the growth inhibitory effects (mm) of coumarin ligands, silver(I) complexes, and adducts synthesised at contents of 5 and 10 µg against *E. coli* and MRSA. For the complexes and adduct 11, a greater inhibitory effect was observed as the amount of compound to which both bacteria were exposed doubled. Silver(I) complexes **3** and **12** and the corresponding NHC adduct of **3**, compound **11**, were both shown to inhibit the growth of *E. coli* at both 5 and 10 µg. When compared to the reference compound SBC3, which possesses the same NHC ligand, they both inhibited the growth of *E. coli* more effectively at both concentrations with zones of inhibition > 6.9 mm. Despite this, the inhibitory effect of the silver(I) complexes and the NHC adduct tested did not compare to the activity of the control antibiotic, tetracycline, at either concentration.

The bis-TPP adduct **5** showed no inhibitory effect against the *E. coli* strain tested, though it showed similar activity against MRSA to the commercially available silver(I) nitrate and silver(II) oxide with zones of inhibition of 7.3 mm and 8.8 mm at concentrations of 5 and 10 µg, respectively, but was less active than the reference compound, SBC3, where the zone of inhibition at 5 µg and 10 µg disk content against *MRSA* were determined to be 10.2 and 12.3 mm respectively. Only silver(I) complex **12** matched the activity of SBC3, with zones of inhibition of 13.4 mm when tested against MRSA. Nevertheless, none of the complexes or adducts tested showed better activity against MRSA than the antibiotic control tetracycline.

In fact, the unsubstituted coumarin silver(I) complex **12** exhibited the best activity against *MRSA*, while it exhibited similar activity against *E. coli* as complex **3** and its corresponding NHC adduct **11**, further indicating that the substituents do not greatly influence the activity of the coumarin scaffold in this case. In contrast, although bis-TPP complex **5** was inactive against *E. coli*, it displayed similar activity against MRSA as its original silver(I) complexes **3**. Similar trends in activity were observed in a study carried out by Tacke et al. [26], where a bis-TPP adduct of a silver(I) benzoate complex was reported to be inactive against an *E. coli* strain, but showed good activity against MRSA.

To obtain a more accurate reflection of the antimicrobial activity of the complexes, the complexes synthesised were assessed by a further series of assays discussed below.

#### 2.3.2. Determination of Antibacterial Activity of Synthesised Coumarin Silver(I) Complexes Using the Broth Microdilution Assay

Using the broth microdilution assay, the antibacterial activity of complexes 2-(2-oxo-2H-chromen-7-yl)- silver(I) complexes **3** and **4**, ligand **2** and TPP adducts **5** and **6**, and control complex **12** were determined against *Escherichia coli* (ATCC-25922), *Pseudomonas aeruginosa* (PA01 ATCCBAA-47), and methicillin-resistant *Staphylococcus aureus* (ATCC-43300) using the EUCAST guidelines [27]. Ligand **1** and coumarin-3-carboxylic acid have been extensively tested by our group for antimicrobial activity, and no activity has been noted [6,7,24]. The lack of definitive structural information regarding the exact mass of the NHC adduct **11** excluded that complex from this study. The relatively poor solubility of the silver(I) complexes in water meant that they were first dissolved in 100% DMSO before being diluted with MHB (Mueller Hinton Broth) to a stock concentration which contained 5% *v*/*v* DMSO. The highest concentration of DMSO present in the test solutions once prepared was 2.05% *v*/*v*; therefore, controls of DMSO from 0–2.05% *v*/*v* prepared in MHB were also tested. The positive and negative antibiotic controls used in this assay were amikacin and erythromycin. The test solutions of erythromycin were prepared using ethanol to aid the solubilisation of the antibiotic. The final concentration of ethanol to which the bacterial strains were exposed was 1.05%, and as with the DMSO control, it was found to have no inhibitory activity on the bacteria. The antibacterial activity of the complexes was initially screened using a single-broth microdilution experiment across a concentration range of 0–1024 µg/mL in triplicate. As precipitation of some of the complexes at a concentration of 256 µg/mL occurred, a final test concentration range of 0–128 µg/mL was determined, and then all silver(I) complexes, their TPP adducts, and controls were tested using the 0–128 µg/mL concentration range. Ligand **2** (Table S4.8) and triphenylphosphine showed no activity against the three strains of bacteria tested, which is common with the previously tested ligand **1** and coumarin-3-carbocylix acid.

The reference antibiotics used in this series of studies were amikacin and erythromycin. First used in the late 1970s, amikacin, a semi-synthetic aminoglycoside antibiotic, is primarily used to treat broad-spectrum bacterial infections [28]. Since its introduction in the clinic, this antibiotic has been listed as one of the most active aminoglycosides against antibiotic-resistant bacteria and remains one of the essential antibiotics used to treat infections caused by multidrug-resistant (MDR) organisms [29,30,31]. The second reference antibiotic used in this study was erythromycin. The broad-spectrum antimicrobial activity of this antibiotic, especially in the treatment of infections caused by gram-positive bacteria resistant to other drugs, was responsible for its popular use in the clinic after its discovery in 1952 [32]. However, due to its low solubility in water and instability in acidic conditions, the widespread use of this antibiotic quickly diminished. Another drawback of its use is the requirement of relatively large doses to elicit a bactericidal effect [33]. Since higher doses of antibiotics have been shown to promote drug resistance, it is no surprise that bacteria have developed multiple resistance mechanisms against the mode of action of this antibiotic [34]. Despite this, erythromycin is still widely used as a bacteriostatic antibiotic drug to treat bacterial infections, which often leads to the need for secondary antibiotic therapies to treat persistent infections [34]. As such, erythromycin was included in this study as a less effective and older antibiotic against the three bacterial strains, *E. coli*, *P. aeruginosa* and MRSA, while amikacin was included as the strong positive antibiotic control.

**Figure 7:**
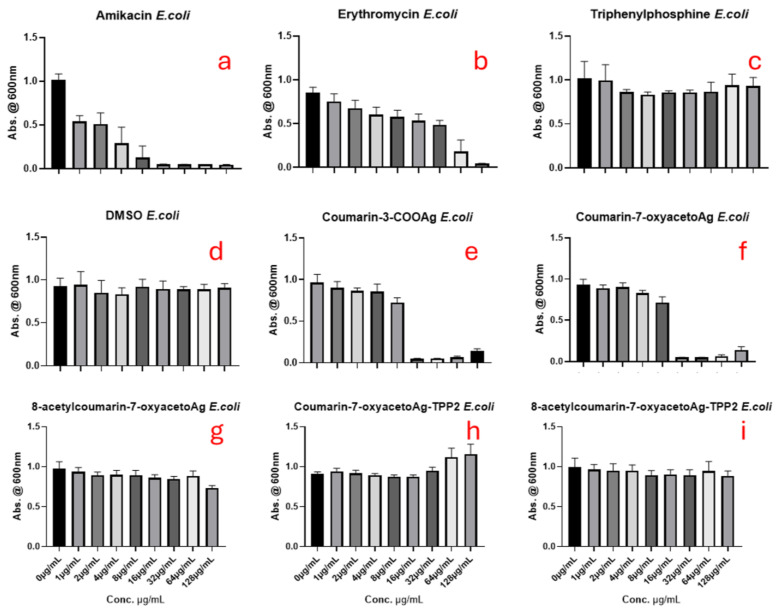
The antimicrobial effects of (**a**) amikacin, (**b**) erythromycin, (**c**) triphenylphosphine (**d**) DMSO, (**e**) coumarin-3-COOAg (**12**), (**f**) coumarin-7-oxyacetoAg (**4**), (**g**) 8-acetylcoumarin-7-oxyacetoAg (**3**), (**h**) coumarin-7-oxyacetoAgTPP2 (**6**), and (**i**) 8-acetylcoumarin-7-oxyacetoAgTPP2 (**5**) on *E. coli* assessed by the micro broth dilution assay.

**Figure 8:**
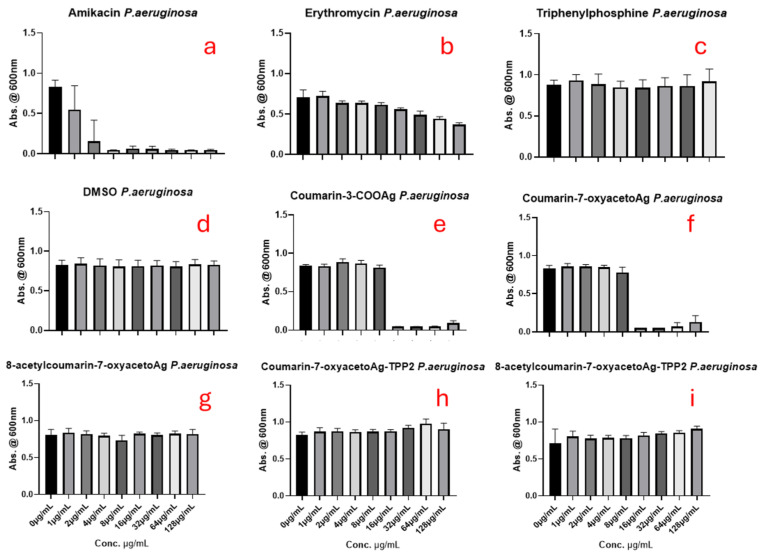
The antimicrobial effects of (**a**) amikacin, (**b**) erythromycin, (**c**) triphenylphosphine (**d**) DMSO, (**e**) coumarin-3-COOAg (**12**), (**f**) coumarin-7-oxyacetoAg (**4**), (**g**) 8-acetylcoumarin-7-oxyacetoAg (**3**), (**h**) coumarin-7-oxyacetoAgTPP2 (**6**) and (**i**) 8-acetylcoumarin-7-oxyacetoAgTPP2 (**5**) on *P. aeruginosa* assessed by the micro broth dilution assay.

**Figure 9:**
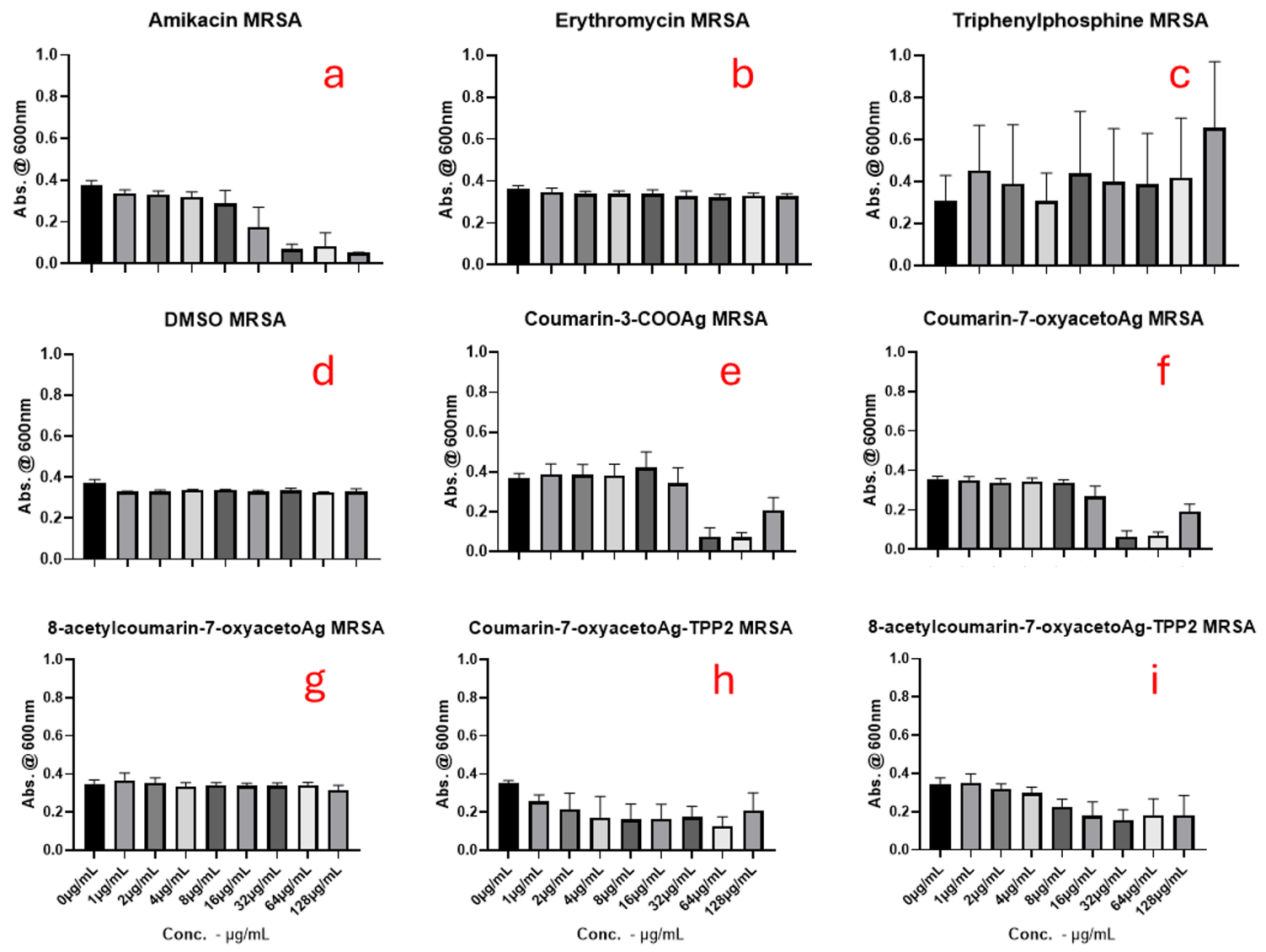
The antimicrobial effects of (**a**) amikacin, (**b**) erythromycin, (**c**) triphenylphosphine (**d**) DMSO, (**e**) coumarin-3-COOAg (**12**), (**f**) coumarin-7-oxyacetoAg (**4**), (**g**) 8-acetylcoumarin-7-oxyacetoAg (**3**), (**h**) coumarin-7-oxyacetoAgTPP2 (**6**) and (**i**) 8-acetylcoumarin-7-oxyacetoAgTPP2 (**5**) on MRSA assessed by the micro broth dilution assay.

Figure 7, Figure 8 and Figure 9 show the results for the antimicrobial screening activity of the complexes. The coumarin-based ligands, ethanol, triphenylphosphine, and DMSO controls showed no activity against any of the bacterial strains tested. In addition, the bis-triphenylphosphino adducts, **5** and **6** tested, were found to have poor activity against the bacterial strains MRSA and *E. coli*, with MIC_50_ values > 88 µg/mL. However, Coumarin-3-COOAg and Coumarin-7-oxyacetoAg compounds had considerable activity at 16 µg/mL and 32 µg/mL, respectively, against *E. coli* and *P. aeruginosa*, as evidenced in Figure 7 and Figure 8 above.

It was noted that complex **3** was inactive against all bacterial strains, which contradicts the findings of a previous study, where **3** had excellent antimicrobial activity against strains of MRSA and *P. aeruginosa* [7]. However, as the strains of bacteria used in this study differed from those used previously to screen **3**, it would appear as if the complex is strain-specific, which is concerning. An additional concern is that this result contradicts the findings of the Kirby Bauer disk diffusion assay, where **3** was found to be the most effective in inhibiting the growth of *E. coli*, indicating that the bacterial growth media may also play a role.

Of all the complexes tested, complex **4** and the reference complex **12** were found to inhibit the growth of the three strains tested most effectively when compared to the untreated control, with activities at higher concentrations comparable to the control amikacin in the case of *E. coli*. The activity of complexes **4** and **12** against MRSA was better than that of the antibiotic control amikacin and, except against *E. coli*, performed better than erythromycin. Overall, these two silver(I) complexes offer excellent therapeutic potential against *E. coli*, *P. aeruginosa*, and MRSA, since their antibacterial activities are better than those of erythromycin and similar to those of amikacin. However, adduct formation, either through the formation of TPP adducts or the NHC carbene adduct, did not significantly improve their antimicrobial activity.

## 3. Materials and Methods

### 3.1. General Experimental Procedures

**Melting point Analysis:** All melting points were recorded on a Stuart Scientific SMP1 melting point apparatus. All values were taken up to 300 °C with a temperature ramp of 0.5 °C per minute and are uncorrected.

**Infrared Spectroscopy:** All samples were prepared as KBr discs, and infrared spectra were recorded in the region of 4000–400 cm^−1^ using an IR Prestige-21 Schimadzu infrared spectrometer (Supplied by Masons Technology Ltd., Dublin, Ireland).

**Nuclear Magnetic Resonance Spectroscopy:** NMR spectra were obtained using a Bruker Advance III 500 MHz spectrometer (Bruker Corporation, Billerica, MA, USA), with all chemical shifts reported as parts per million (ppm, δ). This instrument operated at 500 MHz with a typical resolution of 0.28 Hz for ^1^H NMR and 125 MHz with a typical resolution of 0.45 Hz for ^13^C NMR. All spectra were recorded in either DMSO-d_6_ or CDCl_3_. Signal assignments were aided using standard spectral experiments such as DEPT 90, DEPT 135 and HSQC.

**Elemental Analysis:** The percentage compositions (%C, %H, %N) of some compounds were determined at the Microanalytical laboratory by Ronan Crowley at University College Dublin using an Exeter CE440 elemental analyser (Exeter Analytical, Warwick, UK). Other compounds were analysed by Carmel O’Flaherty and Maryanne Ryan at the Microanalytical laboratory in NUI Maynooth using a Flash EA1112 series NC analyser (ThermoFisher Scientific, Dublin, Ireland), with Eager 300 software.

**X-ray Crystallography:** Crystals **5** and **6** were mounted on a MiTeGen micromount with NVH immersion oil. Data were collected from a shock-cooled single crystal at 150(2) K for **5** and 100(2) K for **6** on a Bruker D8 Quest ECO three-circle diffractometer ((Bruker AXS, Karlsruhe, Germany) with a sealed X-ray tube using graphite as the monochromator and a Bruker PHOTON 50 detector. The diffractometer was equipped with an Oxford Cryostream 800 low-temperature device (Oxford Cryosystems Ltd., Oxford, UK) and used Mo Kα radiation (λ = 0.71073 Å). All data were integrated with SAINT, and multi-scan absorption correction using SADABS was applied [35,36]. The structure was solved by dual methods using SHELXT v2018/2 and refined by full-matrix least-squares methods against F2 by SHELXL using Olex2 [37,38,39]. All non-hydrogen atoms were refined using anisotropic displacement parameters. All hydrogen atoms were refined isotropic on calculated positions using a riding model with their Uiso values constrained to 1.5 times the Ueq of their pivot atoms for terminal sp3 carbon atoms and 1.2 times for all other carbon atoms. The disordered moieties were refined using bond length restraints and displacement parameter restraints.

In **5**, the solvent site is occupied with two different solvents, CH_2_Cl_2_ (13% occupied) and EtOH (12%), with a combined total of 25% occupancy, modelled with geometric (DFIX) and displacement restraints (RIGU, SIMU, ISOR). In **6**, the solvent site CH_2_Cl_2_ is fully occupied.

See Table S4.5 for crystal data and structural details. Crystallographic data for the structures reported here have been deposited at the Cambridge Crystallographic Data Centre [40]. CDC 2396768-2396769 contain the supplementary crystallographic data for this paper. These data can be obtained free of charge from the Cambridge Crystallographic Data Centre via www.ccdc.cam.ac.uk/structures.

**Atomic Absorption Spectroscopy:** The silver content of the synthesised complexes was obtained using an iCE 3000 AA02184306 atomic absorption spectrometer (Agilent Technologies Ireland Ltd., Cork, Ireland) at a wavelength of 328.1 nm with a bandpass width of 0.5 nm. The samples for analysis were digested by boiling in concentrated nitric acid (10 mL) for 1 h. The samples were then diluted with water (50 mL). This stock solution was further diluted to 1/10 and filtered prior to aspiration. Five working standards (2, 3, 5, 7, and 10 ppm) were prepared from a silver standard from Sigma Aldrich (1000 ppm) to generate a calibration curve. All standards and samples were analysed in triplicate.

**Liquid Chromatography/Mass Spectroscopy (LC/MS):** Analysis of compounds was performed using an Agilent Technologies 6530 Accurate Mass Q-TOF LC/MS system (Agilent Technologies Ireland Ltd., Cork, Ireland) tuned in positive mode at 100–2500 M/Z, and data were interpreted using Mass Hunter Software packages-version 2.8. Samples for analysis were dissolved in mobile phase using HPLC-grade solvent and analysed on a C18 Phenomenex column using either acetonitrile: water: 0.1% acetic acid or methanol: water: 0.1% formic acid mobile phases with a flow rate of 0.4 mL/min. The injection volume was set to 1.0 µL. Silver(I) complexes were analysed by continuous infusion using a 50:50 methanol: water mobile phase.

**Reagents and Solvents:** All reagents were purchased from Sigma (Scientific laboratory Supplies, Dublin, Ireland), TCI chemicals (TCI Europe, Zwijndrecht, Belgium), Fisher Scientific (ThermoFisher Scientific, Dublin, Ireland)) or Fluorochem (Fluorochem Eu Ltd., Dublin, Ireland) and used without further purification. All solvents were purchased from Lennox and used again without further purification. LC-MS- and HPLC-grade solvents were purchased from Merck (Scientific laboratory Supplies, Dublin, Ireland). Stocks of anhydrous solvents were prepared by drying over appropriately sized molecular sieves, which were then purged with nitrogen prior to use in the reaction.

### 3.2. Synthesis of [(Bis-triphenylphosphino)-((8-acetyl-2-oxo-2H-chromene-7-yl)oxy]aceto Silver(I) (***5***) [8-AcetylC-oxyaceto-Ag-(TPP)_2_]

To a solution of triphenylphosphine (0.5245 g, 2.00 mmol) in dichloromethane, 8-acetylcoumarin-7-oxyaceto silver(I) (0.369 g, 1.00 mmol) complex was added. The reaction suspension was stirred for 6 h under reflux. Upon cooling, the precipitated solid was filtered under vacuum, washed with cold solvent, and a white-coloured solid was isolated and dried in a vacuum oven at 50 °C for several days. Recrystallisation from DCM/ethanol produced crystals suitable for X-ray crystallographic analysis. **Molecular formula**: C_49_H_39_O_6_AgP_2_; **Yield:** 0.63 g (71%); **M.P.** (°C):141.2–144.5; **^1^H NMR** (DMSO-d_6_) (ppm): 7.93 (d, 1H, *J* = 9.6 Hz, H^4^), 7.50–7.43 (m, 6H, H_TPP_), 7.42 (d, 1H, *J* = 8.8 Hz, H^5^), 7.38–7.27 (m, 25H, H_TPP_), 6.80 (d, 1H, *J* = 8.8 Hz, H^6^), 6.27 (d, 1H *J* = 9.6 Hz, H^3^), 4.52 (s, 2H, H^11^), 2.53 (s, 3H, H^16^); **^13^C NMR** (DMSO-d_6_) (ppm): 198.8 (C^15^), 170.5 (C^12^), 159.6 (C^2^), 158.3 (C^7^), 150.2 (C^9^), 144.4 (C^4^), 132.9–132.8 (d, -CH_ortho_, TPP), 131.6–131.4 (QC, TPP), 129.8 (C^5^), 129.1 (s, -CH_para,_ TPP), 128.4–128.3 (d, -CH_meta_, TPP), 117.8 (C^8^), 111.9 (C^3^), 111.4 (C^10^), 108.9 (C^6^), 67.5 (C^11^), 31.4 (C^16^); **IR** (KBr, cm^−1^): 342, 3054, 1731, 1601, 1479, 1435, 1402, 1353, 1291, 1263, 1229, 1138, 1093, 1027, 997, 833, 744, 694, 515, 501; **CHN** (%): theory: C = 65.86, H = 4.40, Ag = 11.97; found: C = 66.00, H = 4.31, Ag = 12.07.

### 3.3. Synthesis of [Bis-triphenylphosphino)-((2-oxo-2H-chromene-7-yl)oxy]aceto Silver(I) (***6***) [C-7-oxyaceto-Ag-(TPP)_2_]

Using the general synthesis described above, this compound was synthesised using the following reagents: coumarin-7-oxyaceto silver(I) (0.327 g, 1.00 mmol) and triphenylphosphine (0.524 g, 2.00 mmol) with dichloromethane as the reaction solvent. A white-coloured solid was isolated. Crystals suitable for X-ray crystallographic analysis were obtained by the slow diffusion of pentane into dichloromethane. **Molecular formula**: C_47_H_37_O_5_P_2_Ag; **Yield:** 0.66 g (78%); **M.P.** (°C): 139.2–140.1; **^1^H NMR** (DMSO-d_6_) (ppm): 7.96 (d, 1H, *J* = 9.5 Hz, H^4^), 7.53–7.35 (m, 37H, H^5^, H_TPP_), 6.82 (dd, 1H, *J* = 8.7, 2.5 Hz, H^6^), 6.74 (d, 1H, *J* = 2.4 Hz, H^8^), 6.24 (d, 1H, *J* = 9.5 Hz, H^3^), 4.42 (s, 2H, H^11^); **^13^C NMR** (DMSO-d_6_) (ppm): 170.7 (C^12^), 162.3 (C^2^), 160.4 (C^7^), 155.3 (C^9^), 144.4 (C^4^), 133.6–133.5 (d, -CH_ortho_, TPP), 131.9–131.7 (QC, TPP), 130.6 (C^5^), 129.1–129.0 (d, -CH_meta_, TPP), 112.9 (C^6^), 111.9 (C^3^), 111.8 (C^10^), 101.2 (C^8^), 67.9 (C^11^); **IR** (KBr, cm^−1^): 3051, 1722, 1608, 1581, 1505, 1479, 1435, 1404, 1329, 1278, 1227, 1194, 1121, 1095, 1041, 843, 749, 693, 515, 503; **CHN** (%): theory: based on C_47_H_37_O_5_P_2_Ag.CH_2_Cl_2_: C = 61.56, H = 4.20, Ag = 11.52; found: C = 62.59, H = 4.14, Ag = 11.71.

### 3.4. Synthesis of Intermediate Compounds Required for the Isolation of [(1,3-Dibenzyl-4,5-diphenyl imidazole-2-ylidene)–(substituted-2-oxo-2H-chromene-7-yl)]oxyaceto Silver(I) Complexes

#### 3.4.1. General Synthesis of Sodium Salts of 2H-Chromene-2-One Derived Ligands

The appropriately substituted 2*H*-chromene-2-one-derived ligand was stirred in methanol at 50 °C until it was fully dissolved. To this, a methanolic solution of sodium hydroxide was added, with a suspension forming upon addition. The resulting suspension was stirred at 50 °C for 6 h. Upon cooling, the product was isolated via vacuum filtration, washed with cold methanol (2 × 10 mL) followed by cold ethyl acetate (2 × 10 mL) and dried in a vacuum oven at 50 °C for 24 h.

8-Acetylcoumarin-7-Oxyaceto Sodium Salt (**7**) [8-acetyl-C-7-oxyacetoNa]

Using the general synthesis described above, this compound was synthesised using the following reagents: 7-methoxycoumarin-3-carboxylic acid (1.10 g, 5.00 mmol) and sodium hydroxide (0.20 g, 5.00 mmol). An off-white coloured powder was obtained. **Molecular Formula:** C_13_H_9_O_6_Na; **Yield:** 1.06 g, 83%; **M.P.** (°C): >300; **IR** (KBr, cm^−1^): 3482, 3431, 3397, 1728, 1715, 1609, 1557, 1489, 1420, 1402, 1294, 1260, 1225, 1140, 1908, 729, 773, 514; C_13_H_9_O_6_Na.H_2_O: **CHN** (%): theory: C = 51.67, H = 3.19; found: C = 51.63, H = 3.48.

Coumarin-7-Oxyaceto Sodium Salt (**8**) [C-7-oxyacetoNa]

Using the general synthesis described above, this compound was synthesised using the following reagents: coumarin-7-oxyacetic acid (1.50 g, 6.82 mmol) and sodium hydroxide (0.27 g, 6.82 mmol). A white-coloured powder was obtained. **Molecular Formula:** C_11_H_7_O_5_Na; **Yield:** 1.49 g (91%); **M.P.** (°C): >300; **IR** (KBr, cm^−1^): 3438, 3092, 3056, 2949, 2923, 1741, 1617, 1508, 1444, 1429, 1402, 1347, 1283, 1274, 1236, 1201, 1164, 1127, 1055, 995, 838, 821, 688, 619; C_11_H_7_O_5_Na.H_2_O **CHN** (%): theory: C = 50.78, H = 3.49 found: C = 49.69, H = 3.71.

### 3.5. General Synthesis of [(1,3-Dibenzyl-4,5-diphenyl-imidazole-2-ylidene)^+^-(substituted)-(2-oxo-2H-chromene-7-yl)oxy]acetate]^−^ Ionic Intermediates

In a biphasic solution of dichloromethane and deionised water (1:1, 40 mL), the appropriate substituted coumarin sodium salt (1.50 mmol) and [imid Br] were stirred vigorously at 40 °C for 24 h. Upon cooling, the reaction mixture was transferred to a separation funnel, and the dichloromethane layer containing the product was collected. The remaining water layer was extracted with fresh dichloromethane (2 × 10 mL). The dichloromethane layers were collected and pooled together and washed gently with deionised water (2 × 10 mL). The dichloromethane layer was collected, and the solvent was removed under reduced pressure in a water bath heated to 30 °C to obtain a hygroscopic amorphous fluffy solid or oil.

#### 3.5.1. [1,3-Dibenzyl-4,5-diphenylimidazole-2-ylidene)]^+^-[(8-acetylcoumarin-7-oxyacetate)^−^ (***9***) [8-acetylcou-7-oxyacetNHC]

This ionic intermediate was synthesised using the method described above, using the following reagents: [imid Br] (0.4814 g, 1.00 mmol) and 8-acetyl-C-7-oxyacetoNa (0.426 g, 1.50 mmol) A white coloured amorphous solid was obtained. **Molecular Formula:** C_42_H_34_O_6_N_2_; **Yield:** 0.596 g (90%); **^1^H NMR** (DMSO-d_6_) (ppm): 9.72 (s, 1H, H^15^), 7.99 (d, 1H, *J* = 9.5 Hz, H^4^), 7.64 (d, 1H, *J* = 8.7 Hz, H^5^), 7.46–7.26 (m, 18H, H^19/20/23/24^), 7.08 (m, 4H, H^21/25^), 6.90 (d, 1H, *J* = 8.7 Hz, H^6^), 6.28 (d, 1H, *J* = 9.5 Hz, H^3^), 5.42 (s, 4H, H^17^), 4.37 (s, 2H, H^11^), 2.57 (s, 3H, H^14^); **^13^C NMR** (DMSO-d_6_) (ppm): 199.6 (C^13^), 168.3 (C^12^), 159.7 (C^2^), 158.8 (C^7^), 150.2 (C^9^), 144.5 (C^4^), 136.7 (C^15^), 134.1 (C^16^), 131.8 (C^22^), 130.8 (C^19^), 130.2 (C^5^), 129.8 (C^20^), 128.8 (C^24^), 128.5 (C^23^), 127.8 (C^21^), 124.9 (C^18^), 118.3 (C^8^), 112.3 (C^3^), 111.8 (C^10^), 109.8 (C^6^), 68.3 (C^11^), 50.5 (C^17^), 32.1 (C^14^); **IR** (KBr, cm^−1^): 3401, 3059, 3032, 1724, 1601, 1553, 1489, 1456, 1445, 1402, 1534, 1288, 1263, 1226, 1140, 1096, 1020, 930, 837, 764, 7--, 635, 598; **CHN** (%): theory: C = 72.19, H = 5.48, N = 4.01; found: C = 71.85, H = 5.21, N = 4.15.

#### 3.5.2. [1,3-Dibenzyl-4,5-diphenylimidazole-2-ylidene)]^+^-[Coumarin-7-oxyacetate]^−^ (***10***) [cou-7-oxyacetNHC]

This ionic intermediate was synthesised using the method described above using the following reagents: [imid Br] (0.4814 g, 1.00 mmol) and cou-7-oxyacetoNa (0.363 g, 1.5 mmol). A colourless oil was obtained. **Molecular Formula:** C_40_H_32_O_5_N_2_; **Yield:** 0.594 g (96%); **^1^H NMR** (DMSO-d_6_) (ppm): 9.73 (s, 1H, H^15^), 7.96 (d, 1H, *J* = 9.9 Hz, H^4^), 7.57 (d, 1H, *J* = 8.7 Hz, H^5^), 7.45–7.26 (m, 17H, H^19/20/23/24^), 7.12–7.04 (m, 4H, H^21/25^), 6.85 (dd, 1H, *J* = 8.6, 2.4 Hz, H^6^), 6.76 (d, 1H, *J* = 2.5 Hz, H^8^), 6.24 (d, 1H, *J* = 9.5 Hz, H^3^), 5.42 (s, 4H, H^17^), 4.38 (s, 2H, H^11^); **^13^C NMR** (DMSO-d_6_) (ppm): 169.1 (C^12^), 163.1 (C^7^), 160.9 (C^2^), 155.7 (C^9^), 144.9 (C^4^), 137.3 (C^15^) 134.5 (C^16^), 132.3 (C^22^), 131.3 (C^19^), 130.6 (C^20^), 129.5 (C^5^), 129.3 (C^21^), 129.3 (C^25^), 128.9 (C^24^), 128.3 (C^23^), 125.4 (C^18^), 113.3 (C^6^), 112.2 (C^3^), 112.1 (C^10^), 101.7 (C^8^), 68.6 (C^11^), 50.9 (C^17^); **IR** (NaCl plate, cm^−1^): 3049, 1728, 1613, 1554, 1498, 1455, 1446, 1401, 1354, 1335, 1277, 1232, 1192, 1157, 1133, 1123, 1076, 1045, 1021, 979, 928, 892, 839.

### 3.6. Synthesis of [(1,3-Dibenzyl-4,5-diphenyl imidazole-2-ylidene)–(8-acetoxy-2-oxo-2H-chromene-7-oxyaceto)] Silver(I) (***11***) via an Ion Exchange Route

8-acetyl-C-7-oxyacetoNHC intermediate (0.596 g, 0.90 mmol) was dissolved in anhydrous acetonitrile (5 mL), and the solution was degassed with nitrogen. This solution was injected into a flask containing a suspension of silver oxide (0.127 g, 0.55 mmol) in anhydrous acetonitrile, with the resulting suspension stirred under nitrogen in the absence of light at room temperature for 72 h. Following this time, the reaction suspension was filtered by gravity to remove the excess silver oxide. The filtrate was collected and filtered by gravity, followed by filtration through a grade 4 sinter funnel. The final filtrate was collected, and the solvent was removed under reduced pressure in a water bath heated to 35 °C to obtain a light brown-coloured amorphous fluffy solid. **Molecular formula:** C_42_H_33_O_6_N_2_Ag, **Yield**: 0.56 g, 82%; **^1^H NMR:** (500 MHz) (DMSO-d_6_) (ppm): 7.98 (d, 1H, *J* = 9.5 Hz, H^4^), 7.65 (d, 1H, *J* = 8.7 Hz, H^5^), 7.35–7.15 (m, 19H, H^19/21/23/24^), 6.96–6.90 (m, 6H, H^16/21^), 6.29 (d, 1H, *J* = 9.5 Hz, H^3^), 5.33 (s, 4H, H^17^), 4.54 (s, 2H, H^11^), 2.55 (s, 3H, H^14^); **^13^C NMR** (DMSO-d_6_) (ppm): 199.4 (C^13^), 170.6 (C^12^), 159.6 (C^2^), 158.3 (C^7^), 150.2 (C^9^), 144.3 (C^4^), 136.7 (C^15^), 132.2 (C^16^), 130.6 (C^5^), 129.9 (C^23^), 129.2 (C^24^), 128.5 (C^22^), 127.7 (C^19^), 127.5 (C^18^), 126.8 (C^21^), 118.4 (C^8^), 112.6 (C^3^), 112.1 (C^10^), 109.7 (C^6^), 67.9 (C^11^), 52.6 (C^17^), 32.1 (C^14^); **IR** (KBr, cm^−1^): 3061, 3030, 1728, 1601, 1558, 1489, 1447, 1398, 1352, 1287, 1263, 1227, 1140, 1060, 1022, 926, 835, 768, 735, 700; **CHN** (%): theory: C = 65.55 H = 4.32, N = 3.64, Ag = 14.02; found: C = 66.62, H = 4.35, N = 3.87, Ag = 14.42.

### 3.7. Details of Bacterial Strains Used for Biological Studies

The *Escherichia coli* (ATCC-25922) and methicillin-resistant *Staphylococcus aureus* (ATCC-43300) test strains used were obtained from frozen stocks of the collaborative group in UCD, while the *Pseudomonas aeruginosa* (PA01 ATCCBAA-47) test strain was obtained from frozen stocks from the microbiological lab in TU Dublin Tallaght. Fresh frozen stocks of each of the test strains were made by culturing each strain individually in Tryptone Soy Broth (TSB) and storing them with 10% glycerol at −80 °C. Prior to their use in the experiment, they were passaged twice on a Tryptone Soy Agar plate (TSA).

The general method for Kirby Bauer disk diffusion as per the EUCAST guidelines was previously published [24].

#### Preparation of Microtitre Test Plates for the Broth Microdilution Assay

The 96-well microtitre plates were prepared five days prior to the experiment being carried out. Once the plates were loaded with the silver(I) complexes and controls, they were stored in a freezer at −20 °C for a maximum of five days before being used. Prior to being used in the experiment, the plates were thawed in an incubator at 37 °C. The concentration (0–256 µg/mL) of the suspensions of each silver(I) complex was prepared individually and loaded (50 µL) onto a 96-well microtitre plate. Separately, the antibiotic, DMSO and ethanol controls were serially diluted across the microtitre plate with MHB (50 µL) so that concentrations of 0–256 µg/mL were obtained. MHB was also included in each microtitre plate as a negative control in triplicate.

The bacteria test strains were cultured overnight in MHB (10 mL) for 18 h. Fresh MHB (100 mL) was inoculated with the overnight culture, and the bacteria grew to the mid-log phase (OD between 0.6 and 0.8). Once the mid-log phase was reached, the cultures were diluted with MHB to a concentration of 1 × 10^6^ CFU/mL and loaded onto the microtitre plates already containing the complexes. The final volume in each well was 100 µL, the final concentration of the inoculum was 5 × 10^5^ CFU/mL, and the final test concentration range obtained was 0–128 µg/mL. The plates were then incubated in a stack of three plates in an aerobic environment at 37 °C with agitation (~65 rpm) for 24 h. After this time, the microtitre plates were analysed on a multi-plate reader at an absorbance of 600 nm. The average OD at 600 nm for each complex tested was obtained, and the results are presented in the following section. Replicate experiments (n = 3) were carried out on independent days with independent overnight cultures of the test strains of bacteria. Six replicates of each complex were tested, giving a total of eighteen readings per complex, while the antibiotics and controls were tested in triplicate, giving a total of nine readings each.

## 4. Conclusions

This work detailed the synthesis and characterisation of heteroleptic complexes of coumarin, firstly the bis-triphenylphosphino adducts of acetoxy substituted and unsubstituted coumarin-7-oxyaceto silver(I) complexes **5** and **6**. As the bis-TPP methoxy substituted coumarin-3-carboxylato silver(I) adducts were isolated previously, the bis-TPP coumarin-7-oxyaceto silver (I) adducts displayed an overall increased solubility profile when compared to the parent silver(I) complexes and relatively good photostability in DMSO-d_6_. The antimicrobial activity of the complexes was assessed via two different techniques, the Kirby Bauer disc diffusion assay and the broth microdilution assay, with the introduction of the TPP ligands reducing the activity of the complexes against *E. Coli* and *P. aeruginosa* in the case of the coumarin-7-oxyaceto derivative in both cases. All of these studies were actually prompted by the previously reported antimicrobial activity of the 8-acetylcoumarin-7-oxyacetosilver(I) complex [7], but the activity of that complex was very limited in these studies. Our previous work was carried out using different strains of these pathogenic bacteria; nevertheless, the results were surprising.

Analysis of **11** by ^1^H and ^13^C NMR spectroscopy, IR spectroscopy, microanalysis, and AAS indicated that this complex was likely formed as a complex aggregate species with an overall stoichiometry of 1:1:1 coumarin:Ag(I):NHC. However, despite forming these complex aggregates, the silver(I) adduct exhibited better photostability and increased solubility when compared to the parent silver(I) complex. Our ultimate goal of achieving water solubility was not achieved in this case. The Kirby Bauer method was used to determine the antimicrobial activity of the adduct complex, with the heteroleptic complex formation not adversely affecting its activity against *E. coli* and MRSA. Indeed, given its likely large molecular mass, the adduct complex has good antimicrobial activity at low concentrations, but our lack of a clear structural definition of the complex’s exact mass would likely hinder its development for further studies as a systemic therapeutic.

In future work, exploring the potential of combining these novel compounds with established antibiotics, such as erythromycin, could be a promising avenue to develop effective adjunct or combination therapies, enhancing their antimicrobial efficacy and addressing complex bacterial resistance mechanisms.

## Figures and Tables

**Table 1:** Main IR bands (cm^−1^), melting points (°C) and microanalytical data of adducts **5** and **6.**

Complex	Main IR Bands (cm^−1^)					Melting Point (°C)		
	ν_CO_ δ Lactone	ν_asym_ (OCO)	ν_sym_ (OCO)					
[8-acetyl-C-7-oxyacetoAg(TPP_2_)] (**5**)	1731	1479	1353			141.2–144.5		
[C-7-oxyacetoAg(TPP_2_)] (**6**)	1722	1479	1329			139.2–140.1		
**Microanalytical and AAS data**								
**Complex**	**Molecular Formula**		**Theory (%)**			**Found (%)**		
			**C**	**H**	**Ag**	**C**	**H**	**Ag**
[8-acetyl-C-7-oxyaceto Ag(TPP_2_)] (**5**)	C_49_H_39_O_6_AgP_2_		65.86	4.40	11.97	66.00	4.31	12.07
[C-7-oxyacetoAg(TPP_2_)] (**6**)	C_47_H_37_O_5_AgP_2_.CH_2_Cl_2_		61.56	4.20	11.52	62.59	4.14	11.71

**Table 2:** Selected ^1^H and ^13^C NMR spectral data (ppm) for compounds **3**, **4** (R = Ag(I)), and **5**, **6** (R = Ag-TPP_2_) recorded in DMSO-d_6_.

Derivative	(8-Acetyl) = R^1^				(H) = R^1^			
	H^5^	H^11^	C^11^	C^12^	H^5^	H^11^	C^11^	C^12^
**R^2^ = Ag(I)** **(3, 4)**	7.66	4.60	68.0	170.0	7.58	4.54	67.5	170.8
**R^2^= Ag-TPP_2_** **(5, 6)**	7.42	4.49	67.5	170.5	7.53–7.35	4.42	67.9	170.7

**Table 3:** Selected bond lengths (Å) and angles of [bis-triphenylphosphino)-(substituted-2-oxo-2H-chromen-7-oxyaceto]silver(I) adducts **5** and **6**.

Bond Lengths [Å]		
	(**5**)	(**6**)
**Ag(1)-P(1)**	2.4060(8)	2.4104(6)
**Ag(1)-P(2)**	2.4317(8)	2.4040(6)
**Ag(1)-O(1)**	2.383(2)	2.4548(16)
**Ag(1)-O(3)**	2.538(2)	2.4061(16)
**O(1)-C(2)**	1.248(4)	1.261(3)
**O(3)-C(2)**	1.248(4)	1.250(3)
**Bond Angles (^°^)**		
	(**5**)	(**6**)
**P(1)-Ag(1)-O(1)**	115.94(6)	116.40(4)
**P(2)-Ag(1)-P(1)**	127.53(3)	126.62(2)
**P(2)-Ag(1)-O(1)**	114.57(6)	111.08(4)
**P(2)-Ag(1)-O(3)**	98.19(6)	114.06(4)
**O(3)-Ag(I)-P(1)**	122.81(6)	112.63(4)
**O(3)-Ag(1)-O(1)**	53.51(7)	54.51(5)
**O(3)-C(2)-O(1)**	125.65(3)	125.0(2)

**Table 4:** Hydrogen bond geometries (Å and °) observed in bis-triphenylphosphino)-(substituted-2-oxo-2H-chromen-7-oxyaceto]silver(I) adducts **5** and **6.**

Hydrogen Bonds for 5 (Å and °)				
**D-H…A**	**d(D-H)**	**d(H…A)**	**d(D…A)**	**<(DHA)**
**C(29)-H(29)…O(1)#1**	0.95	2.53	3.293(4)	137.6
**C(34)-H(34)…O(3)#2**	0.95	2.45	3.177(4)	132.9
**C(40)-H(40)…O(17)#3**	0.95	2.65	3.236(4)	120.4
**C(41)-H(41)…O(17)#3**	0.95	2.64	3.229(5)	120.6
**Hydrogen Bonds for 6 (** **Å and °)**				
**C(33)-H(33)…O(3)#1**	0.95	2.56	3.150(3)	121
**C(43)-H(43)…O(9)**	0.95	2.55	3.156(3)	122

Symmetry transformations used to generate equivalent atoms for **5**: #1 –x + 1, −y + 1, −z = 1; #2 − x + 1, −y + 1, −z; #3 x + 1, y + 1, z; **6**: #1 x − 1, y, z.

**Table 5:** Yield (%), main IR bands and microanalytical data of compounds **7–10**.

Compound	Yield (%)	Main IR Bands (cm^−1^)								
		ν_CO_ δ Lactone	ν_asym_ (OCO)		ν_sym_ (OCO)			ν_C-N_ (imid)	ν_C-C_ (imid)	
[8-acetyl-C-7-oxyacetoNa] (7)	83	1728	1609		1294					
[C-7-oxyacetoNa] (8)	91	1741	1617		1283					
[8-acetylcou-7-oxyacetNHC] (9)	90	1724	1601		1288			1553	1456	
[cou-7-oxyacetNHC] (10)	96	1728	1613		1277			1554	1455	
[(8-acetyl-C-7-oxyaceto)(Ag)(NHC)] (11)	82	1728	1601		1398			1558	1489	
**Microanalytical Data**										
**Compound**	**Molecular Formula**		**Theory (%)**				**Found (%)**			
			**C**	**H**	**N**	**Ag**	**C**	**H**	**N**	**Ag**
[8-acetyl-C-7-oxyacetoNa] (7)	C_13_H_9_O_6_Na·H_2_O		51.67	3.67			51.63	3.48		
[C-7-oxyacetoNa] (8)	C_11_H_7_O_5_Na·H_2_O		50.78	3.49			49.69	3.71		
[8-acetyl-C-7-oxyacetoNHC] (9)	C_42_H_34_O_6_N_2_·(H_2_O)_2_		72.19	5.48	4.01		71.85	5.21	4.15	
[(8-acetyl-C-7-oxyaceto)(Ag)(NHC)] (11)	C_42_H_33_O_6_N_2_Ag		65.55	4.32	3.64	14.02	66.42	4.35	3.80	14.42

**Table 6:** Comparison of ^1^H and ^13^C NMR spectral data for compounds **9** and **10** and precursors **1** and **2** and **imidBr**, showing the chemical shifts (ppm) of H^5^, H^11^, C^11^ and C^12^ recorded in DMSO-d_6_.

Derivative	Oxyacetic Acid				NHC Ionic Intermediate			
	H^15^	H^11^	C^11^	C^12^	H^15^	H^11^	C^11^	C^12^
**8-acetylcou-7-oxyacet**	N/A	4.91	65.1	169.5	9.72	4.37	68.3	168.3
**cou-7-oxyacet**	N/A	4.83	64.8	169.6	9.73	4.38	68.6	169.1
**imidBr**	9.64	N/A	N/A	N/A				

N/A: not applicable.

**Table 7:** Average diameter (mm) of the zone of inhibition of test compounds at 5 µg and 10 µg in DMSO (100%) against *E. coli* and MRSA.

Compound	Average Zone of Inhibition Against *E. coli* (diam. mm)		Average Zone of Inhibition Against MRSA (diam. mm)	
	5 µg	10 µg	5 µg	10 µg
**1** and **coumarin-3 carboxylic acid**	Inactive		Inactive	
[8-acetyl-C-7-oxyacetoAg(I)] (**3**)	8.5 ± 0.3	9.1 ± 0.5	7.6 ± 0.5	8.4 ± 0.5
[8-acetylcou-7-oxyacetoAgTPP_2_] (**5**)	Inactive	Inactive	7.3 ± 0.6	8.8 ± 0.9
[8-acetylcou-7-oxyacetoAgNHC] (**11**)	7.6± 0.6	8.6 ± 0.5	8.0 ± 0.7	10.1 ± 0.9
[Coumarin-3-COOAg(I)] (**12**)	6.7 ± 0.6	8.3 ± 0.4	7.3 ± 0.7	13.4 ± 1.0
**Tetracycline**	15.7 ± 1.1	18.0 ± 0.9	19.4 ± 0.5	21.9 ± 1.1
**SBC3**	6.9 ± 0.8	7.7 ± 0.6	10.2 ± 1.7	12.3 ± 2.1
[ImidBr]	2.9 ± 4.1	7.7 ± 1.3	9.6 ± 0.6	12.2 ± 1.0
Silver nitrate	8.9 ± 0.2	9.7 ± 0.7	7.9 ±0.2	8.8 ± 0.6
Silver oxide	8.2 ± 0.8	9.2 ± 0.5	7.6 ± 0.5	8.3 ± 0.4
Triphenylphosphine	Inactive	Inactive	Inactive	Inactive
DMSO (100%)	Inactive	Inactive	Inactive	Inactive

## Data Availability

The raw data supporting the conclusions of this article will be made available by the authors on request.

## Supplementary Materials

The following supporting information can be downloaded at: https://www.mdpi.com/article/10.3390/molecules29245917/s1. Scheme S1: Overall synthetic scheme for isolation of ligands **1** and **2** and complexes **3** and **4.** Figure S1.1: Numbering system used for the assignment of 1H and 13C NMR spectra of 7-acetyl-2-(2-oxo- 2H-chromene-2-one). Figure S1.2: Numbering system used for the assignment of 1H and 13C NMR spectra of 8-acetyl-7-hydroxy- (2-oxo-2H-chromene-2-one). Figure S1.3: Numbering system used for the assignment of 1H and 13C NMR spectra of ethyl 2-(2-oxo-2H-chromene-substituted-yl)oxy acetates, where R = -COCH3 and -CO = C15 and -CH3 = C16. Figure S1.4: Numbering system used for the assignment of 1H and 13C NMR spectra of 2-(2-oxo-2H-chromen-substituted-yl)oxy acetic acid ligands and their corresponding silver(I) complexes, where R = -COCH3 and -CO = C15 and -CH3 = C16. Figure S1.5: Numbering system used for the assignment of 1H spectra of 1,3-dibenzyl-4,5-diphenyl-imidazolium bromide. Figure S3.1 (a): 1H NMR spectrum of [8acetyl-C-7-oxyaceto Ag(TPP2)] (**5**); recorded in DMSO-d6. Figure S3.1(b): 1H NMR spectrum of [C-7-oxyaceto Ag(TPP2)] (**6**); recorded in DMSO-d6. Figure S3.2a: 1H NMR solution photostability study of [8acetyl-C-7-oxyacetoAg(TPP2)] (**5**) in the presence of UV/Vis light over a 96 h period in DMSO-d6. Figure S3.2b: 1H NMR solution photostability study of [8acetyl-C-7-oxyacetoAg(TPP2)] (**5**) in the absence of UV/Vis light over a 96 h period in DMSO-d6. Figure S3.2c: 1H NMR solution photostability study of [C-7-oxyacetoAg(TPP2)] (**6**) in the *absence* of UV/Vis light over a 96 h period in DMSO-d6. Figure S3.2d: 1H NMR solution photostability study of [C-7-oxyacetoAg(TPP2)] (**6**) in the *presence* of UV/Vis light over a 96 h period in DMSO-d6. Figure S3.3a. Molecular structure of [bis-triphenylphosphino)-(8-acetyl-2-oxo-2H-chromen-7-oxyaceto]silver(I) (**5**) showing disordered the disordered solvent site with two different solvents, CH2Cl2 (13% occupied) and EtOH (12%), combined total 25% occupancy. Displacement shown at 50% probability. Heteroatoms labelled only. Figure S3.3b. Molecular structure of [bis-triphenylphosphino)-(8-acetyl-2-oxo-2H-chromen-7-oxyaceto]silver(I) (**5**) showing H-bonding interactions. Figure S3.4a Molecular structure of [bis-triphenylphosphino)-(2-oxo-2H-chromen-7-oxyaceto]silver(I) (**6**) showing the fully occupied CH2Cl2 solvent site. Displacement shown at 50% probability. Heteroatoms labelled only. Figure S3.4b Molecular structure of [bis-triphenylphosphino)-(2-oxo-2H-chromen-7-oxyaceto]silver(I) (**6**) showing H-bonding interactions. Figure S3.5: 1H NMR spectrum of [|(8-acetylcou-7-oxyacet)(NHC)] (**10**) recorded in DMSO-d6. Figure S3.6 a: Stability of **11** examined in DMSO-d6 (absence of light). Figure S3.6 b: Stability of **11** examined in DMSO-d6 (presence of UV/Vis light). Figure S3.6 c: Stability of **11** examined in CDCl3 (absence of light). Figure S3.6 d: Stability of **11** examined in CDCl3 (presence of UV/Vis light). Table S4.1: Compound names and abbreviations. Table S4.2: Experimental yields (%), melting points (°C ) and main IR bands of compounds **1–4**. Table S4.3: Experimental and literature values of **1–6**, showing chemical shifts (ppm), multiplicity patterns and *J* values (Hz); recorded in DMSO-d6; OacetH gp. (oxyacetic acid group) = -OCH2COOH; Acet. gp (acetyl group) = -COOCH3. Table S4.4: 13C NMR data of **1–6** showing the chemical shifts (ppm) recorded in DMSO-d6. Table S4.5: Crystallographic and structural refinement data for **5** and **6**. Table S4.6: NMR spectral data for compounds **9–11** showing the chemical shifts (ppm), multiplicity patterns and J-values (Hz); recorded in DMSO-d6; *n/o = not observed. Table S4.7: Table of MS Data for Adduct 11. Table S4.8. Growth Curves for ligand **2** against *E. coli*, MRSA and *P. aeruginosa*, measured using the Micro Broth Dilution Assay.
