# Supplementary material for: Heteroleptic Coumarin-Based Silver(I) Complexes: Possible New Antimicrobial Agents
Source: Molecules. 2024 Dec 15;29(24):5917. doi: 10.3390/molecules29245917 (PMC11678713; doi:10.3390/molecules29245917)
Supplement: Supplementary file 1 [file molecules-29-05917-s001.zip › molecules-3336829-supplementary.pdf]

**Supplementary information :** Heteroleptic coumarin-based silver(I) complexes: possible new antimicrobial agents

Erika Mooney,[1,5] Brendan Twamley,[2] Gordon Cooke,[1,5] Emma Caraher,[1,5] Matthias Tacke,[3] Fintan Kelleher,[1,5] Bernadette S. Creaven.[4,5]

1. *School of Chemical and BioPharmaceutical Sciences, Technological University Dublin, TU Dublin, Tallaght Campus, D24 FKT9, Ireland.*
2. *School of Chemistry, Trinity College Dublin.*
3. *UCD School of Chemistry, Science Centre South, University College Dublin, Belfield, Dublin 4 .*
4. *School of Chemical and BioPharmaceutical Sciences, Technological University Dublin, Central Quad Building, Grangegorman, Dublin D07 ADY7*
5. Centre for AMR and One Health Research, *Technological University Dublin, TU Dublin, Tallaght Campus, D24 FKT9, Ireland.*

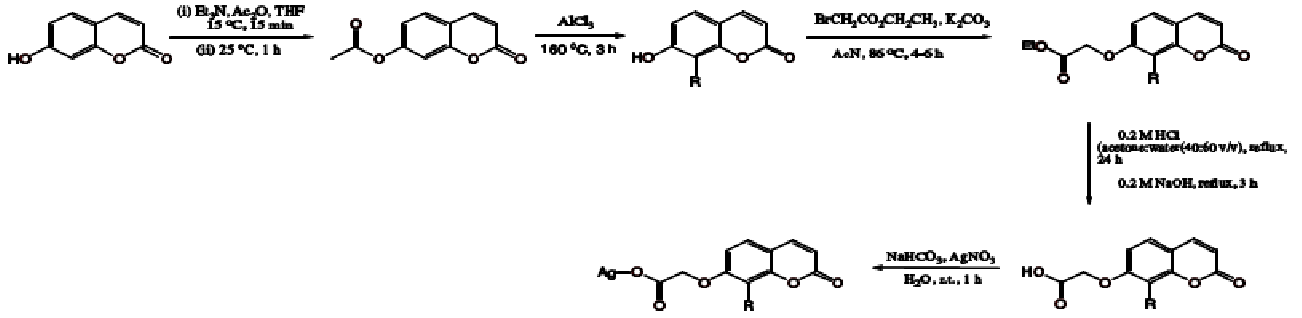


3,4

1,2

Scheme S1: Overall synthetic scheme for isolation of ligands **1** and **2** and complexes **3** and **4**

**S.1.1: Synthesis of 7-acetoxy-2-(2-oxo-2H-chromene-2-one) [7-acetoxycou]**

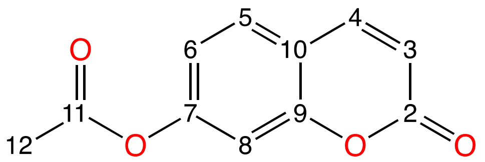

**Figure S1.1:** Numbering system used for the assignment of $^{1}H$ and $^{13}C$ NMR spectra of 7-acetyl-2-(2-oxo-2H-chromene-2-one)

7-Hydroxycoumarin (1.00 g, 6.20 mmol) was added to tetrahydrofuran (25 mL). The resulting canary yellow coloured suspension was cooled in an ice-bath to 15 °C. Upon cooling, triethylamine (2.044 g, 2.82 mL, 20.20 mmol) was added. Following dissolution of the 7-hydroxycoumarin, acetic anhydride (1.24 g, 1.13 mL, 12 mmol) was added dropwise over 10 min so that the temperature of the solution was maintained at 15 °C. The resulting solution was slowly brought to room temperature and stirred for 1 h. After this time, removal of tetrahydrofuran under reduced pressure afforded a white coloured solid which was then recrystallised from hot ethanol. The resulting colourless needle-like solid was collected via vacuum filtration and dried in a vacuum oven at 50 °C for several hours. **Molecular Formula:** $C_{11}H_8O_4$; **Yield:** 1.27 g (49%); **M.P.** (°C): 134-137 [Lit. 142-144];[183] $\mathbf{R_f}$: 0.71 (80:20, ethyl acetate:hexane); $\mathbf{^{1}H}$ **NMR** (DMSO-$d_6$)(ppm): 8.07 (d, 1H, *J* = 9.5 Hz, $H^4$), 7.77 (d, 1H, *J* = 8.5 Hz, $H^5$), 7.27 (s, 1H, $H^8$), 7.16 (d, 1H, *J* = 8.4 Hz, $H^6$), 6.48 (d, 1H, *J* = 9.5 Hz, $H^3$), 2.31 (s, 3H, $H^{12}$); $\mathbf{^{13}C}$ **NMR** (DMSO-$d_6$)(ppm): 168.8 ($C^{11}$), 159.7 ($C^2$), 154.1 ($C^7$), 152.9 ($C^9$), 143.8 ($C^4$), 129.3 ($C^5$), 118.7 ($C^6$), 116.7 ($C^{10}$), 115.5 ($C^3$), 110.1 ($C^8$), 20.9 ($C^{12}$); **IR** (KBr, $cm^{-1}$): 3120, 3081, 3057, 2945, 1768, 1740, 1621, 1566, 1507, 1428, 1400, 1373, 1333, 1269, 1234, 1199, 1186, 1123, 1012, 989, 907, 849, 824, 760, 750, 726, 641, 619, 603, 587, 513, 450.

**S.1.2: Synthesis of 8-acetyl-7-hydroxy-(2-oxo-2H-chromene-2-one) [8-acetyl-7-OHcou]**

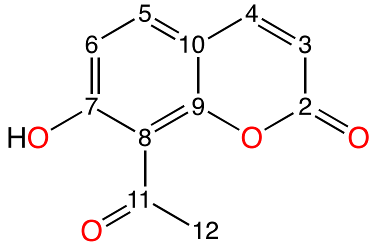

**Figure S1.2:** Numbering system used for the assignment of $^{1}H$ and $^{13}C$ NMR spectra of 8-acetyl-7-hydroxy-(2-oxo-2H-chromene-2-one)

7-Acetylcoumarin (2.00 g, 9.79 mmol) and aluminium chloride powder (5.97 g, 44.79 mmol) were heated at 160 °C for 3 h. The resulting viscous red coloured oil was cooled to room temperature. Cooling over an ice-bath, ice-cold water (20 mL) was added dropwise to the red-coloured solid. A white coloured solid began to form and following addition of water the resulting suspension was stirred for 1 h at room temperature. The product was collected via vacuum filtration and recrystallised from hot ethyl acetate (15 mL). The resulting yellow coloured crystalline solid was recovered via vacuum filtration and dried in a vacuum oven at 50 °C for several hours. **Molecular Formula:**$C_{11}H_8O_4$; **Yield:** 2.0 g (54%); **M.P.** (°C): 158.3-162.7 [Lit. 166-168];[183] $\mathbf{R_f}$: 0.74 (80:20, ethyl acetate:hexane); $\mathbf{^{1}H}$ **NMR** (DMSO-$d_6$)(ppm): 11.77 (s, 1H, -OH), 7.99 (d, 1H, *J* = 9.6 Hz, $H^4$), 7.67 (d, 1H, *J* = 8.7 Hz, $H^5$), 6.91 (d, 1H, *J* = 8.6 Hz, $H^6$), 6.29 (d, 1H, *J* = 9.5 Hz, $H^3$), 2.31 (s, 3H, $H^{12}$); $\mathbf{^{13}C}$ **NMR** (DMSO-$d_6$)(ppm): 200.7 ($C^7$), 159.9 ($C^2$), 159.4 ($C^{11}$), 152.5 ($C^9$), 144.7 ($C^4$), 131.9 ($C^5$), 114.7 ($C^8$), 113.5 ($C^6$), 111.8 ($C^3$), 111.2 ($C^{10}$), 32.5 ($C^{12}$); **IR** (KBr, $cm^{-1}$): 1739, 1627, 1616, 1594, 1568, 1411, 1369, 136, 1329, 1289, 1235, 1214, 1165, 1121, 1098, 1055, 1031, 980, 862, 847, 778, 714, 675, 648, 630, 554, 477.

**S.1.3: General synthesis of ethyl 2-(2-oxo-2H-chromene-substituted-yl) oxy acetates**

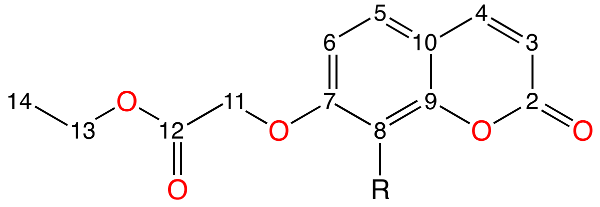


**Figure S1.3:** Numbering system used for the assignment of $^{1}H$ and $^{13}C$ NMR spectra of ethyl 2-(2-oxo-2H-chromene-substituted-yl)oxy acetates, where R = $-COCH_3$ and -<u>C</u>O = $C^{15}$ and -<u>C</u>$H_3$ = $C^{16}$

To a suspension of anhydrous potassium carbonate (1.0 mmol) in anhydrous acetonitrile (20 mL) was added the appropriately substituted coumarin-7-hydroxycoumarin (1.0 mmol). The reaction mixture was heated to reflux and stirred for 20 min. Then, ethyl bromoacetate (1.0 mmol) was added in a single portion and the reaction stirred under nitrogen for 4-6 h. Upon completion of the reaction as indicated by TLC using an 80:20 hexane: ethyl acetate mobile phase, the reaction mixture was cooled. Upon cooling, excess potassium carbonate was removed via vacuum filtration. The filtrate was collected, and acetonitrile removed under reduced pressure to obtain a pale-yellow solid. The crude product was triturated with hexane to obtain a white coloured crystalline solid which was then collected and dried in a vacuum oven at 50 $^{\circ C}$ for several hours.

**S.1.3.1: Ethyl-2-[(8-acetyl-2-oxo-2H-chromene-7-yl) oxy]acetate [8acetyl-C-oxyacet-Et]**

Using the general synthesis outlined above, this compound was synthesised using the following reagents: 8-acetyl-7-OHcou (0.816 g, 4.00 mmol), anhydrous potassium carbonate (0.552 g, 4.00 mmol), ethyl bromoacetate (0.44 mL, 4.00 mmol) and acetonitrile (40 mL). A deep yellow coloured crystalline solid was obtained. **Molecular Formula:**$C_{15}H_{14}O_6$; **Yield:** 0.963 g (83%); **M.P.** ($^{\circ C}$):105-107 [Lit. 105-107];[129] **$R_f$:** 0.58 (80:20 ethyl acetate:hexane); **$^{1}$H NMR** (DMSO-$d_6$)(ppm): 8.02 (d, 1H, *J* = 9.7 Hz, $H^4$), 7.74 (d, 1H, *J* = 8.8 Hz, $H^5$), 7.12 (d, 1H, *J* = 8.8 Hz, $H^6$), 6.37 (d, 1H, *J* = 9.6 Hz, $H^3$), 5.02 (s, 2H, $H^{11}$), 4.17 (q, 2H, *J* = 7.2 Hz, $H^{13}$), 2.56 (s, 3H, $H^{16}$), 1.21 (t, 3H, *J* = 7.1 Hz, $H^{14}$); **$^{13}$C NMR** (DMSO-$d_6$)(ppm): 198.8 ($C^{12}$), 168.0 ($C^2$), 159.3 ($C^{15}$), 156.5 ($C^7$), 150.2 ($C^9$), 144.1 ($C^4$), 130.3 ($C^5$), 118.7 ($C^8$), 113.5 ($C^3$), 113.2 ($C^{10}$), 109.3 ($C^6$), 65.3 ($C^{11}$), 60.9 ($C^{13}$), 32.1 ($C^{16}$), 14.0 ($C^{14}$); **IR** (KBr, $cm^{-1}$): 3075, 2991, 1737, 1720, 1600, 1568, 1485,1454, 1421, 1406, 1379, 1354, 1300, 1278, 1246, 1207, 1172, 1143, 1120, 1103, 1037, 977, 941, 846, 808, 775, 731, 696, 673, 603, 509.

**S1.3.2: Ethyl-2-[(2-oxo-2H-chromene-7-yl)oxy]acetate [C-7oxyacet-Et]**

Using the general synthesis outlined above, this compound was synthesised using the following reagents: 7-hydroxycoumarin (1.29 g 8.00 mmol), anhydrous potassium carbonate (1.106 g, 8.00 mmol), ethyl bromoacetate (1.34g, 0.885 mL, 8.00 mmol) and acetonitrile (80 mL). A white coloured solid was obtained. **Molecular Formula:** $C_{13}H_{12}O_5$; **Yield:** 1.77 g (89%); **M.P.** ($^{\circ C}$): 104.2-107.5 [Lit. 107-110];[185] **$R_f$:** 0.77 (80:20 ethyl acetate: hexane); **$^{1}$H NMR** (DMSO-$d_6$)(ppm): 7.99 (d, 1H, *J* = 9.5 Hz, $H^4$), 7.64 (d, 1H, *J* = 8.5 Hz, $H^5$), 6.99 (d, 1H, *J* = 2.5 Hz, $H^8$), 6.97 (dd, 1H, *J* = 8.6, 2.5 Hz, $H^6$), 6.32 (d, 1H, *J* = 9.6 Hz, $H^3$), 4.92 (s, 2H, $H^{11}$), 4.18 (q, 2H, *J* = 7.2 Hz, $H^{13}$), 1.22 (t, 3H, *J* = 7.2 Hz, $H^{14}$); **$^{13}$C NMR** (DMSO-$d_6$)(ppm): 168.2 ($C^{12}$), 160.7 ($C^2$), 160.2 ($C^7$), 155.2 ($C^9$), 144.2 ($C^4$), 129.5 ($C^5$), 112.9 ($C^6$), 112.8 ($C^{10}$), 112.6 ($C^3$), 101.5 ($C^8$), 64.9 ($C^{11}$), 60.8 ($C^{13}$), 14.0 ($C^{14}$); **IR** (KBr, $cm^{-1}$): 3076, 2990, 2955, 1746, 1724, 1711, 1612, 1560, 1509, 1456, 1400, 1377, 1358, 1350, 1285, 1264, 1222, 1194, 1157, 1125, 1069, 1018, 981, 897, 863, 844, 755, 616, 477.

### S.1.4 General synthesis of 2-(2-oxo-2H-chromen-substituted-yl)oxy acetic acid ligands

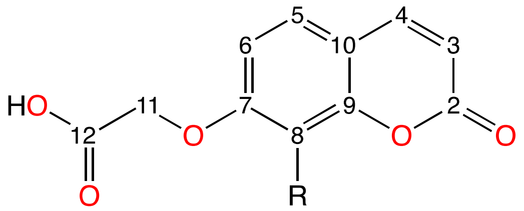

**Figure S1.4:** Numbering system used for the assignment of $^1$H and $^{13}$C NMR spectra of 2-(2-oxo-2H-chromen-substituted-yl)oxy acetic acid ligands and their corresponding silver(I) complexes, where R = -$COCH_3$ and -<u>C</u>O = $C^{15}$ and -<u>C</u>$H_3$ = $C^{16}$

The appropriate ethyl 2-(2-oxo-2H-chromene-7-yl)oxy acetate was suspended in a 10% v/v hydrochloric acid solution (50 mL) [acetone:water (40:60) mixture]. The reaction mixture was stirred at reflux temperature for 24 h. Upon reaction completion as indicated by TLC (80:20; chloroform:methanol) the reaction solution was allowed to cool and a colourless crystalline solid formed. The precipitate was isolated via vacuum filtration, washed with cold deionised water (20 mL) and dried in a vacuum oven for several hours.

#### S.1.4.1 2-[(8-acetyl-2-oxo-2H-chromene-7-yl)oxy]acetic acid (1) [8acetyl-C-oxyacetH]

This compound was synthesised by hydrolysing the ester group of 8acetyl-C-oxyacet-Et (0.291 g, 1.00 mmol) to the corresponding acid, using the general synthesis described above A colourless crystalline solid was obtained. **Molecular Formula:** $C_{13}H_{10}O_6$; **Yield:** 0.161 g (62%); **M.P.** ($^{\circ C}$): 217.0-221.0 [Lit. 217-219][129]; **$^1$H NMR** (DMSO-$d_6$)(ppm): 13.24 (s, 1H, -OH), 8.02 (d, 1H, *J* = 9.7 Hz, $H^4$), 7.73 (d, 1H, *J* = 8.6 Hz, $H^5$), 7.09 (d, 1H, *J* = 8.8 Hz, $H^6$), 6.35 (d, 1H, *J* = 9.6 Hz, $H^3$), 4.91 (s, 2H, $H^{11}$), 2.55(s, 3H, $H^{16}$); **$^{13}$C NMR** (DMSO-$d_6$)(ppm): 198.8 ($C^{12}$), 169.5 ($C^2$), 159.4 ($C^{13}$), 156.7 ($C^7$), 150.3 ($C^9$), 144.1 ($C^4$), 130.2 ($C^5$), 118.7 ($C^8$), 113.4 ($C^3$), 113.1 ($C^{10}$), 109.2 ($C^6$), 65.1 ($C^{11}$), 32.1 ($C^{14}$); **IR** (KBr, $cm^{-1}$): 3071, 2956, 2908, 1761, 1693, 1670, 1597, 1564, 1492, 1440, 1406, 1359, 1307, 1259, 1207, 1176, 1157, 1116, 1002, 941, 916, 840, 813, 775, 738, 678, 659, 601, 580.

#### S.1.4.2: 2-[(2-oxo-2H-chromene-7-yl)oxy]acetic acid (2) [C-7oxyacetH]

An adapted method was used to synthesise this compound. C-7oxyacet-Et (0.50 g, 2.01 mmol) was stirred in a 10% aqueous sodium hydroxide solution at reflux temperature for 3 h. Upon cooling, the solution was acidified with 2 M hydrochloric acid to precipitate the product as a white coloured solid. The precipitated product was collected via vacuum filtration and washed with cold deionised. The resulting white coloured powder was dried in a vacuum oven at 50 $^{\circ C}$ for several days. **Molecular Formula:**$C_{11}H_8O_5$; **Yield:** 0.37 g (84%); **M.P.** ($^{\circ C}$): 216.8-219.2 [Lit. 220-223]; **$^1$H NMR** (DMSO-$d_6$)(ppm): 13.14 (s, 1H, -OH), 7.99 (d, 1H, *J* = 9.4 Hz, $H^4$), 7.64 (m, 1H,$H^5$), 6.97-6.95 (m, 2H, $H^{6/8}$), 6.30 (d, 1H, *J* = 9.5 Hz, $H^3$), 4.83 (s, 2H, $H^{11}$); **$^{13}$C NMR** (DMSO-$d_6$)(ppm): 169.6 ($C^{12}$), 160.9 ($C^2$), 160.2 ($C^7$), 155.2 ($C^9$), 144.3 ($C^4$), 129.5 ($C^5$), 112.8 ($C^{10}$), 112.7 ($C^6$), 112.6 ($C^3$), 101.5 ($C^8$), 64.8 ($C^{11}$); **IR** (KBr, $cm^{-1}$): 3078, 3001, 2965, 2911, 2794, 2736, 2593, 1730, 1619, 1561, 1511, 1458, 1420, 1403, 1350, 1286, 1252, 1229, 1215, 1156, 1125, 1074, 999, 895, 860, 835, 766, 751, 729, 671, 617, 518, 462.

### S.1.5 Synthesis of silver(I) complexes of 2-(2-oxo-2H-chromene-substituted-yl)oxy acetic acid derived ligands

The silver(I) complexes derived from 2-(2-oxo-2H-7-yl)oxy acetic acid were synthesised by dissolving the appropriate ligand (1.00 mmol) in deionised water (10 mL) which contained sodium hydrogen carbonate (1.00 mmol). Once fully dissolved, a solution of silver(I) nitrate (1.00 mmol) in deionised water (10 mL) was added and upon addition a white coloured precipitate formed immediately. The resulting suspension was stirred at room temperature in the absence of light for 1 h. After this time the product was collect via vacuum filtration and washed with cold deionised water (2 x 10 mL), collected and dried in a vacuum oven at 50 $^{\circ C}$ in the absence of light for several days.

**S.1.5.1 2-[(8-acetyl-2-oxo-2H-chromene-7-yl)oxy]aceto Ag(I) (3) [(8acetyl-C-7-oxyaceto)Ag]**

Using the general synthesis described above this complex was synthesised using the following reagents: compound **1** (0.49 g, 1.87 mmol), sodium hydrogen carbonate (0.157 g, 1.87 mmol), silver(I) nitrate (0.318 g, 1.87 mmol) and deionised water (20 mL). A white coloured powder was obtained. **Molecular Formula:** $C_{13}H_9O_6Ag$; **Yield:** 0.34 g (50%); **M.P.** (°C ): [Lit.];[129] **$^1$H NMR** (DMSO-d$_6$)(ppm): **$^1$H NMR** (DMSO-d$_6$)(ppm): 7.99 (d, 1H, *J* = 9.6 Hz, H$^4$), 7.65 (d, 1H, *J* = 8.8 Hz, H$^5$), 6.95 (d, 1H, *J* = 8.8 Hz, H$^8$), 6.30 (d, 1H, *J* = 9.5 Hz, H$^3$), 4.59 (s, 2H, H$^{11}$), 2.57 (s, 3H, H$^{14}$); **$^{13}$C NMR** (DMSO-d$_6$)(ppm): 199.4 (C$^{13}$), 170.5 (C$^{12}$), 159.6 (C$^2$), 158.3 (C$^7$), 150.2 (C$^9$), 144.4 (C$^4$), 129.9 (C$^5$), 118.5 (C$^8$), 112.7 (C$^3$), 112.2 (C$^{10}$), 109.7 (C$^6$), 68.0 (C$^{11}$), 32.2 (C$^{14}$); **IR** (KBr, cm$^{-1}$): 3053, 3077, 3046, 1719, 1644, 1602, 1492, 1418, 1359, 1298, 1255, 1141, 1089, 1015, 955, 938, 832, 774, 743, 698.

**S.1.5.2: 2-[(2-oxo-2H-chromene-7-yl)oxy]aceto Ag(I) (4) [(C-7-oxyaceto)Ag]**

Using the general synthesis described above, this complex was synthesised using the following reagents: compound **2** (0.50 g, 2.26 mmol), sodium hydrogen carbonate (0.189 g, 2.26 mmol), silver(I) nitrate (0.384 g, 2.26 mmol) and deionised water (20 mL). A white coloured powder was obtained. **Molecular Formula:**$C_{11}H_7O_5Ag$; **Yield:** 0.53 g (72%); **M.P.** (°C ): 270.2-273.3; **$^1$H NMR** (DMSO-d$_6$)(ppm): 7.97 (d, 1H, *J* = 9.5 Hz, H$^4$), 7.58 (d, 1H, *J* = 8.7 Hz, H$^5$), 6.88 (dd, 1H, *J* = 8.7, 2.4 Hz, H$^6$), 6.80 (d, 1H, *J* = 2.5 Hz, H$^8$), 6.25 (d, 1H *J* = 9.5 Hz, H$^3$), 4.54 (s, 2H, H$^{11}$); **$^{13}$C NMR** (DMSO-d$_6$)(ppm): 170.8 (C$^{12}$), 161.9 (C$^2$), 160.3 (C$^7$), 155.2 (C$^9$), 144.4 (C$^4$), 129.2 (C$^5$), 112.8 (C$^6$), 112.1 (C$^3$), 112.0 (C$^{10}$), 101.3 (C$^8$), 67.5 (C$^{11}$); **IR** (KBr, cm$^{-1}$): 1727, 1710, 1613, 1421, 1346, 1278, 1232, 1199, 1128, 1055, 993, 831.

**S.1.6 Synthesis of 1,3-dibenzyl-4,5-diphenyl-imidazolium bromide [imidBr]**

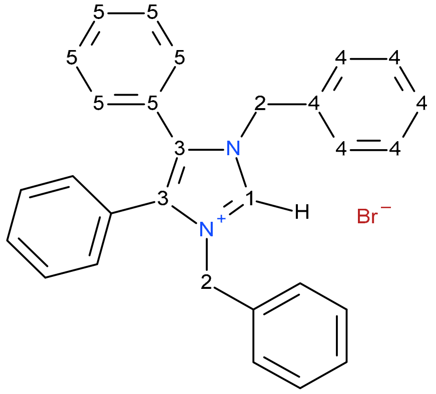


**Figure S1.5:** Numbering system used for the assignment of $^1$H spectra of 1,3-dibenzyl-4,5-diphenyl-imidazolium bromide

4,5-Diphenyl-1*H*-imidazole (2.20 g, 10 mmol) was dissolved in acetonitrile (60 mL). To this solution, potassium carbonate (2.07 g, 15 mmol) and benzyl bromide (2.62 mL, 22 mmol) were added. The resulting suspension was stirred at 35 °C for 72 h. Following 72 h, the precipitate was removed via vacuum filtration. The filtrate was collected and the solvent removed under reduced pressure to yield a white solid. This solid was re-suspended in 5 mL of acetonitrile and the final product isolated through the addition of diethyl ether (100 mL). The final white coloured solid was isolated via vacuum filtration, washed with diethyl ether (2 x 10 mL) and dried in a vacuum oven at 50 °C for 24 h. **Molecular Formula:** $C_{29}H_{25}N_2Br$; **Yield:** 3.75 g, 78%; **M.P.** (°C ): 214.0-215.3; **$^1$H NMR** (DMSO-d$_6$)(ppm): 9.64 (s, 1H, H$^1$), 7.44-7.08 Ar-H (m, 20H, H$^4$/ H$^5$), 5.42 (s, 1H, H$^2$); **IR** (KBr, cm$^{-1}$): 3053, 3030, 3001, 2931, 1556, 1452, 1352, 1211, 1178, 1157, 1074, 1024, 756, 731, 700, 696, 646, 592, 513.

**S.2 General method for Kirby Bauer Disk Diffusion as per EUCAST guidelines**

Mueller Hinton agar (MHA) was prepared according to manufacturer's instructions and agar plates were prepared with a uniform depth for each plate. Once poured, the agar plates were inverted and stored in a fridge at 4 $^{o}$C. Before use, the plates were air-dried in a laminated airflow cabinet until no visible water droplets remained on the agar.

Mueller Hinton broth (MHB) (10 mL) was inoculated with the bacterium and incubated overnight with agitation (180 rpm) in an aerobic environment for 18 h. After this time, fresh MHB (100 mL) was inoculated with the overnight culture (10 mL) and the bacterium suspension grown to mid-log phase or an optical density (OD) of 0.6 to 0.8. Using a growth curve, the bacterium was diluted with MHB to a concentration of $1x10^{8}$ colony forming units per mL (CFU/mL). Within 60 min of the bacterium suspension being prepared, 50 µL was loaded onto each agar plate and spread evenly using a sterile hockey stick to create a lawn. When the bacteria lawn was prepared on each plate, within 15 min, the disks loaded with either silver(I) complexes, ligands or antibiotic controls were placed firmly and evenly around the plate. Whatman paper disks were evenly placed into four marked quadrants. Stock solutions of ligands, silver oxide, silver(I) complexes and control antibiotics were made to a 1 mg/mL dilution in DMSO. Two volumes of 5 µL and 10 µL were tested for sample against both bacterial strains. DMSO, without the addition of a silver complex, was used as a control in 5 µL and 10 µL volumes. Once the disks were placed onto the agar plates, the agar plates were inverted and incubated in a stack of three at 35 $^{o}$C for 18 h before the diameter (mm) of the zones of inhibition were measured with a callipers. Replicate experiments (n=3) using independent overnight cultures was carried out so that each complex was tested in triplicate per experiment. Thus, a total of nine readings per compound were obtained.

## S.3 Figures

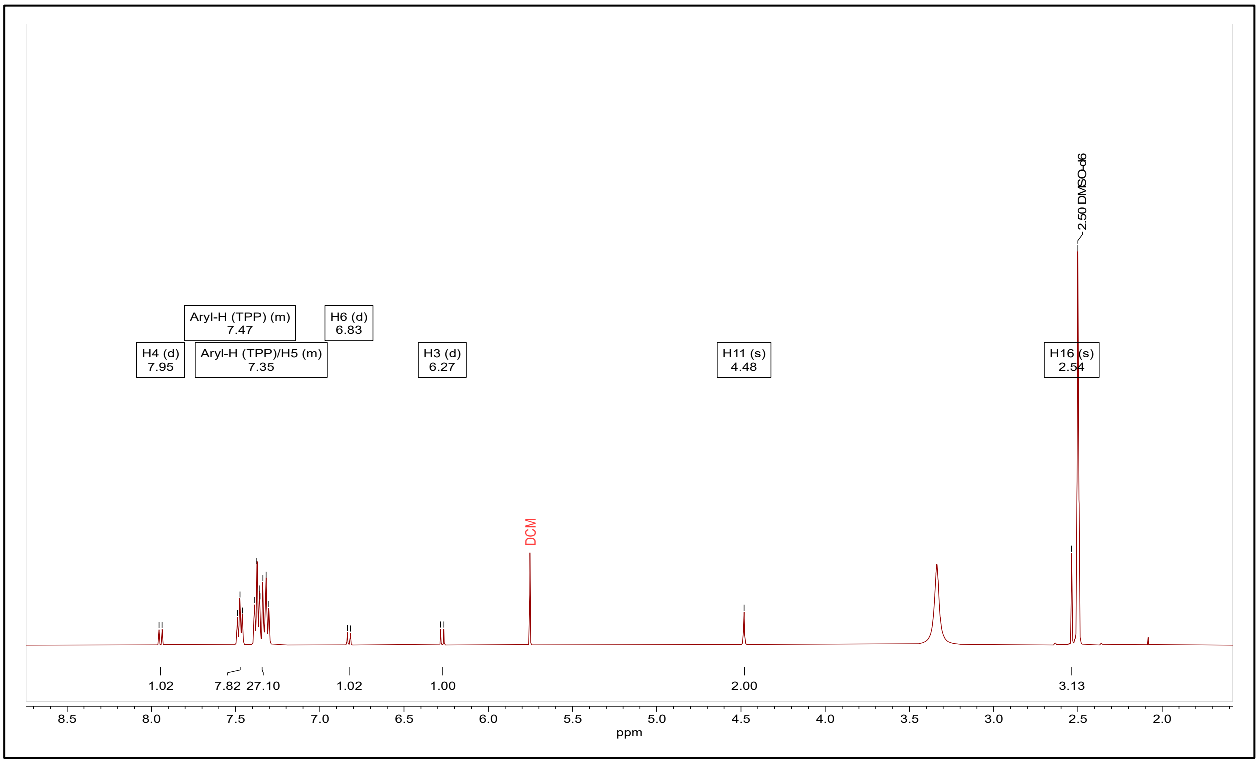


**Figure S3.1 (a):** $^{1}$H NMR spectrum of [8acetyl-C-7-oxyaceto Ag($TPP_2$)] (**5**); recorded in DMSO-$d_6$

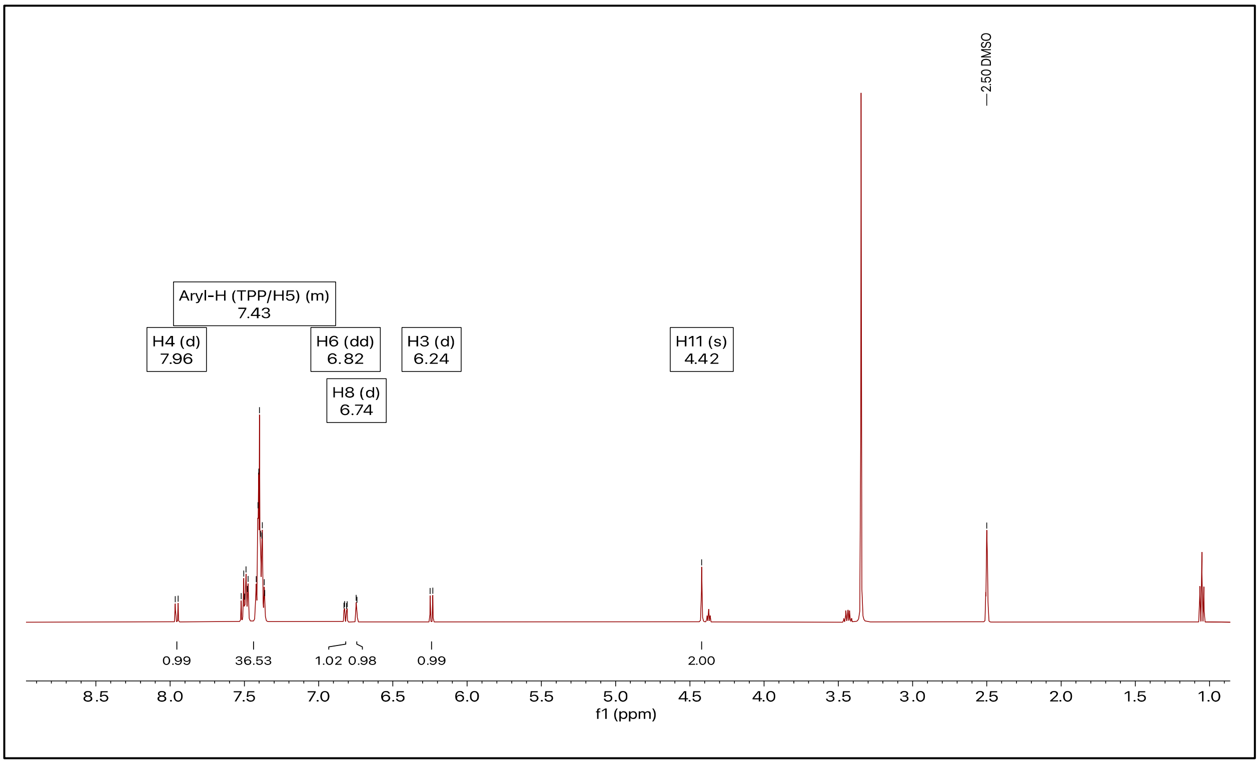


**Figure S3.1 (b):** $^{1}$H NMR spectrum of [C-7-oxyaceto Ag($TPP_2$)] (**6**); recorded in DMSO-$d_6$

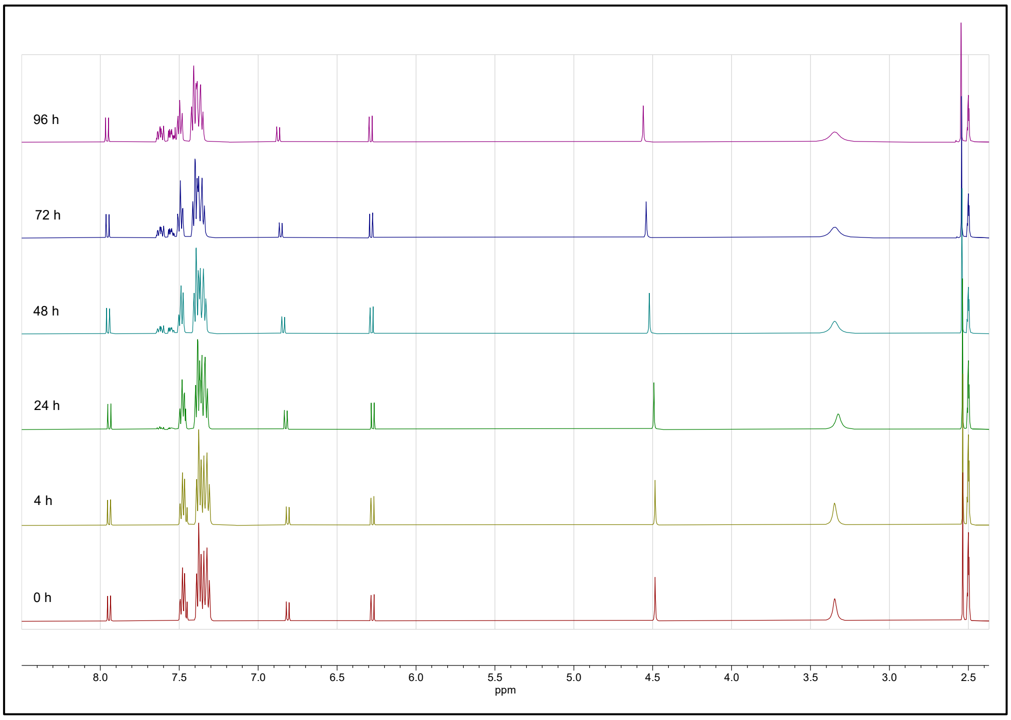


**Figure 3.1a:** $^{1}$H NMR solution photostability study of [8acetyl-C-7-oxyacetoAg(TPP$_2$)] (**5**) in the presence of UV/Vis light over a 96 h period in DMSO-d$_6$

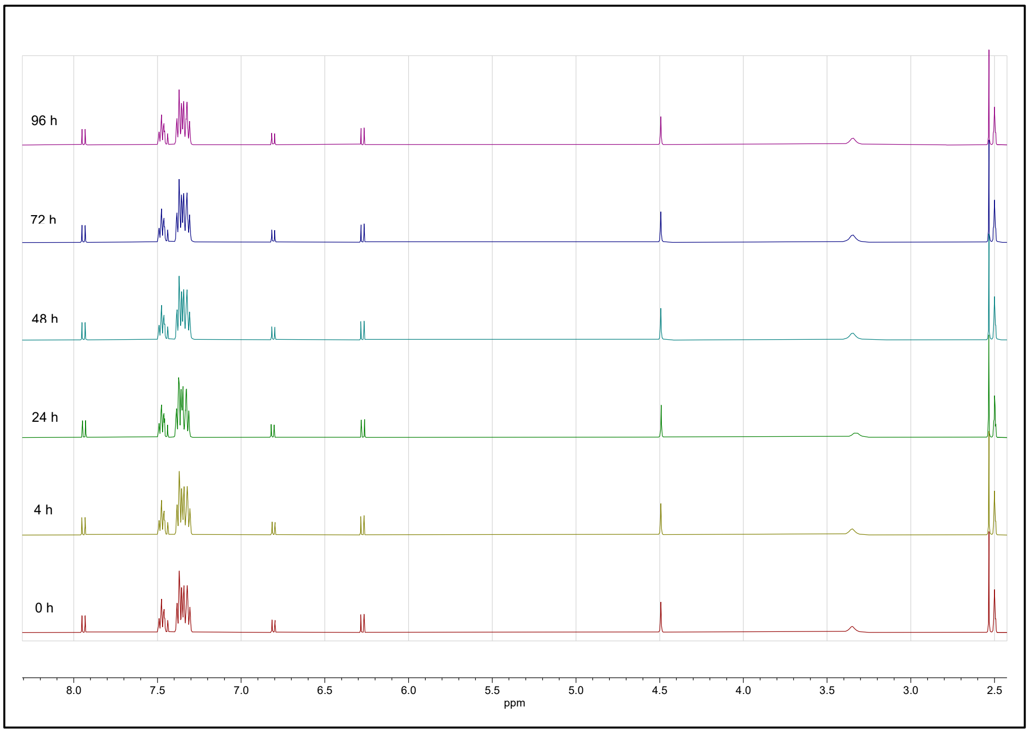


**Figure 3.2b:** $^{1}$H NMR solution photostability study of [8acetyl-C-7-oxyacetoAg(TPP$_2$)] (**5**) in the absence of UV/Vis light over a 96 h period in DMSO-d$_6$

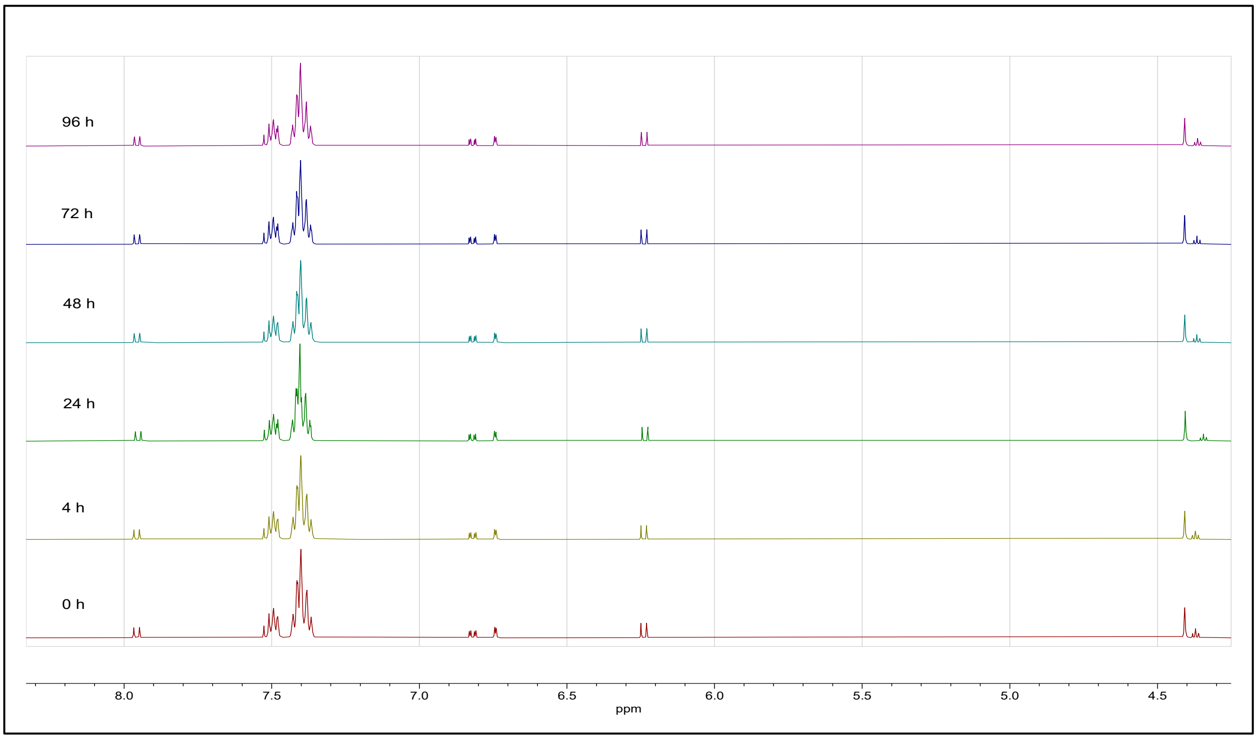


**Figure 3.3c:** $^{1}H$ NMR solution photostability study of [C-7-oxyacetoAg(TPP$_2$)] (**6**) in the *absence* of UV/*Vis* light over a 96 h period in DMSO-$d_6$

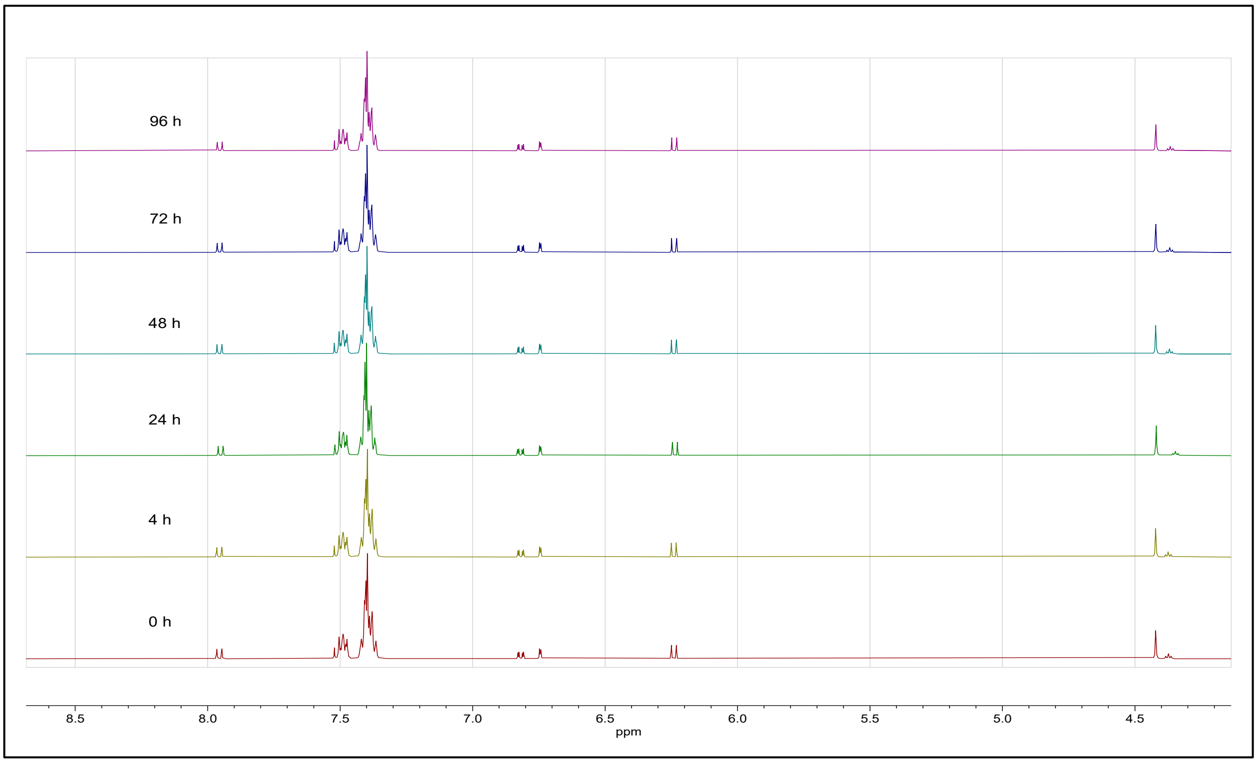


**Figure 3.4d:** $^{1}H$ NMR solution photostability study of [C-7-oxyacetoAg(TPP$_2$)] (**6**) in the *presence* of UV/*Vis* light over a 96 h period in DMSO-$d_6$

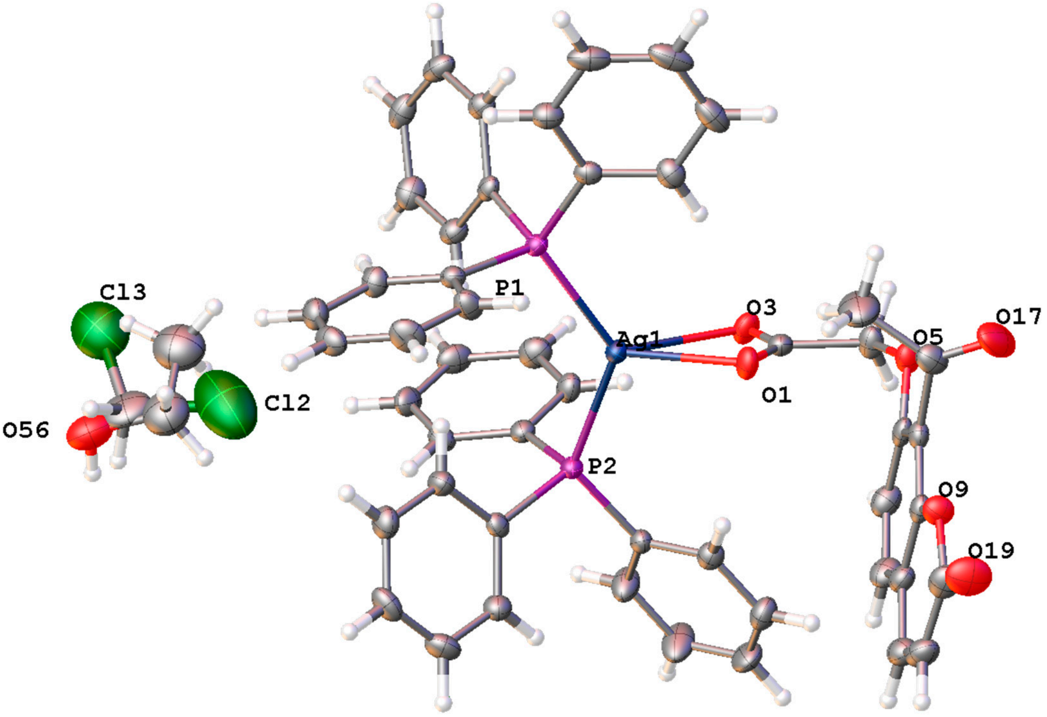


**Figure S3.3a.** Molecular structure of [bis-triphenylphosphino)-(8-acetyl-2-oxo-2H-chromen-7-oxyaceto]silver(I) (**5**) showing disordered the disordered solvent site with two different solvents, $CH_2Cl_2$ (13% occupied) and EtOH (12%), combined total 25% occupancy. Displacement shown at 50% probability. Heteroatoms labelled only.

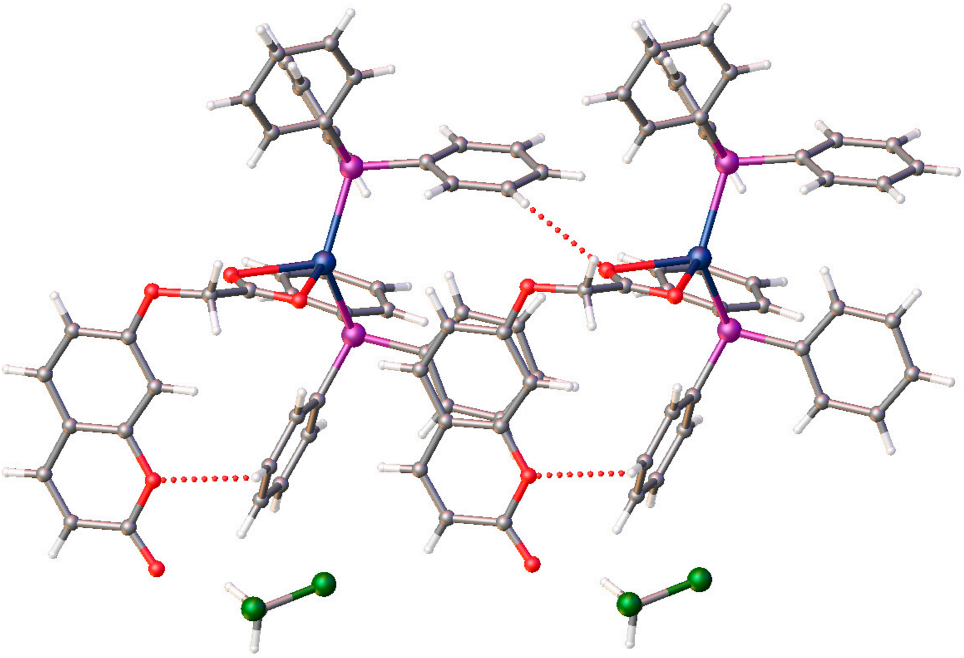

**Figure S3.3b.** Molecular structure of [bis-triphenylphosphino)-(8-acetyl-2-oxo-2H-chromen-7-oxyaceto]silver(I) (**5**) showing H-bonding interactions.

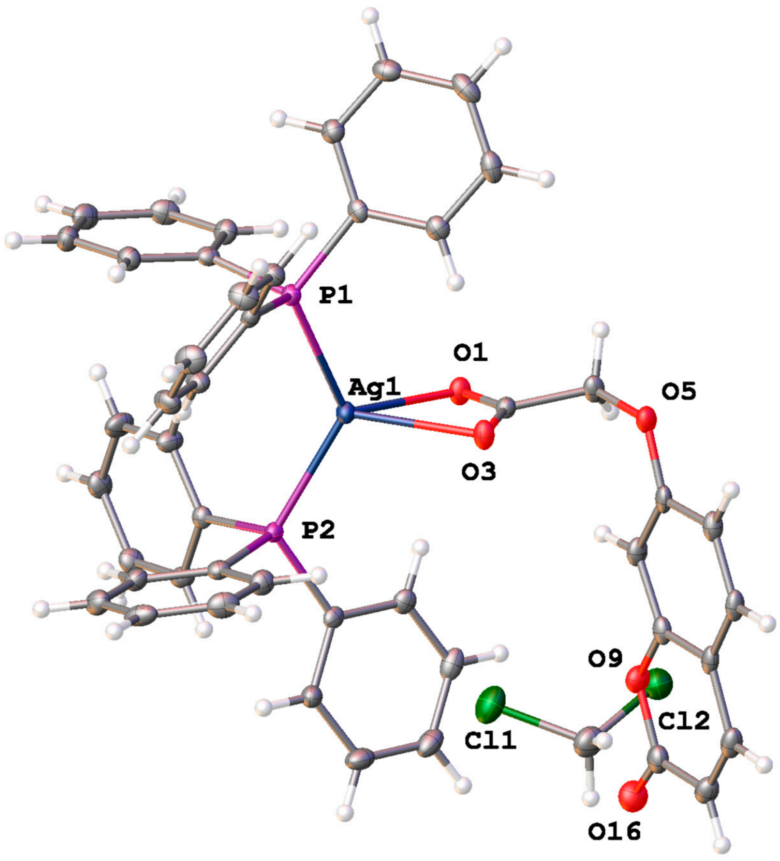


**Figure S3.4a** Molecular structure of [bis-triphenylphosphino)-(2-oxo-2H-chromen-7-oxyaceto]silver(I) (**6**) showing the fully occupied $CH_2Cl_2$ solvent site . Displacement shown at 50% probability. Heteroatoms labelled only.

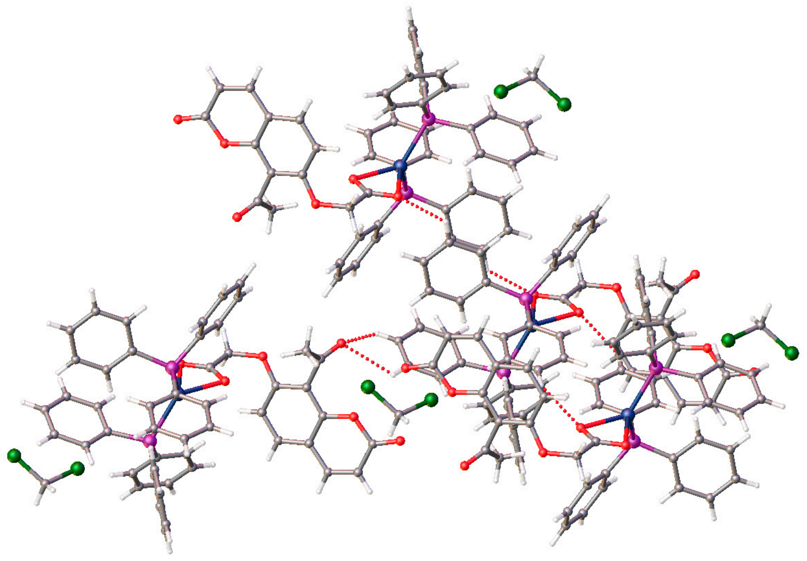

**Figure S3.4b** Molecular structure of [bis-triphenylphosphino)-(2-oxo-2H-chromen-7-oxyaceto]silver(I) (**6**) showing H-bonding interactions.

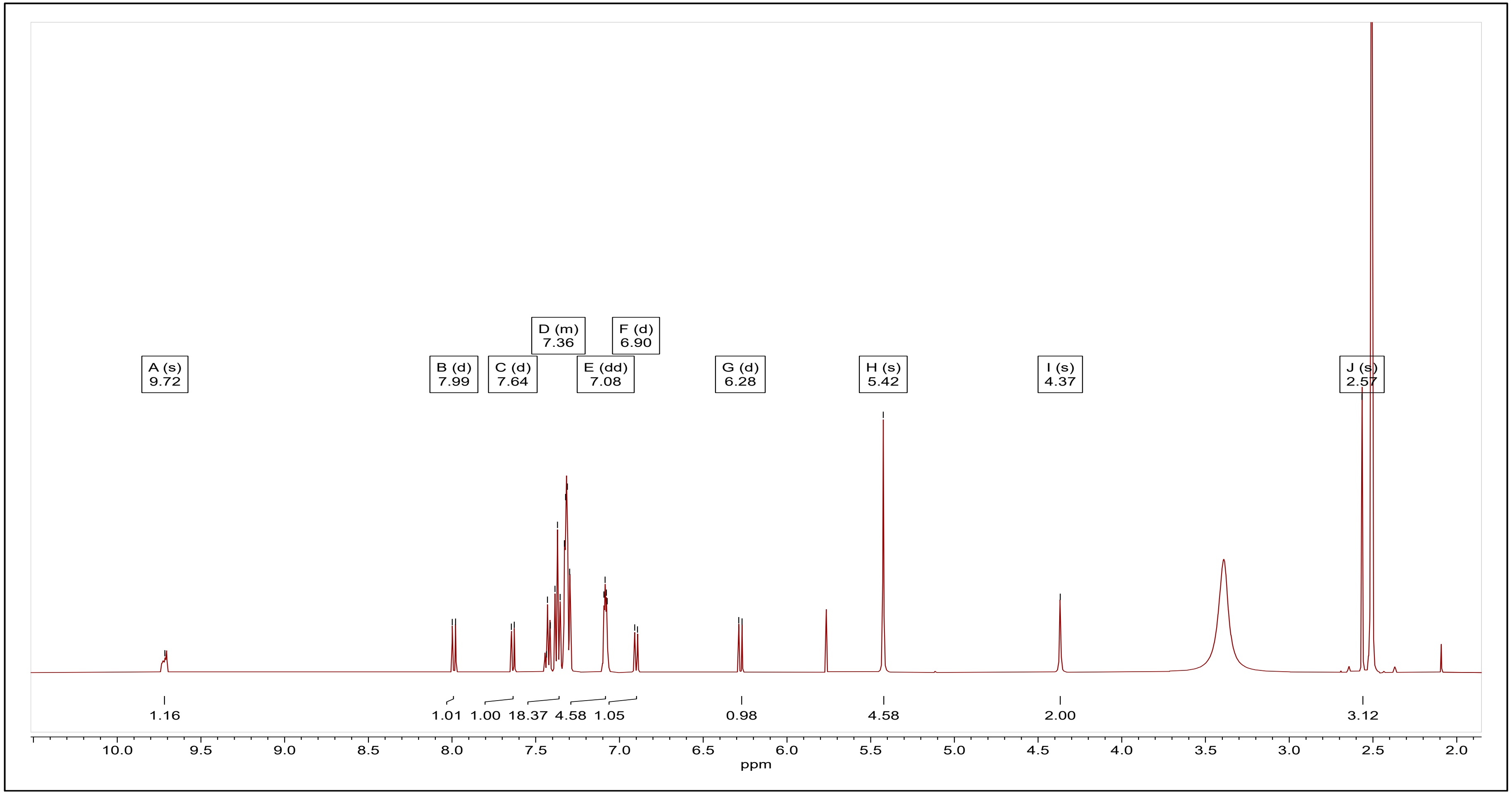


**Figure S3.5:** $^{1}$H NMR spectrum of [|(8-acetylcou-7-oxyacet)(NHC)] (**10**) recorded in DMSO-$d_6$.

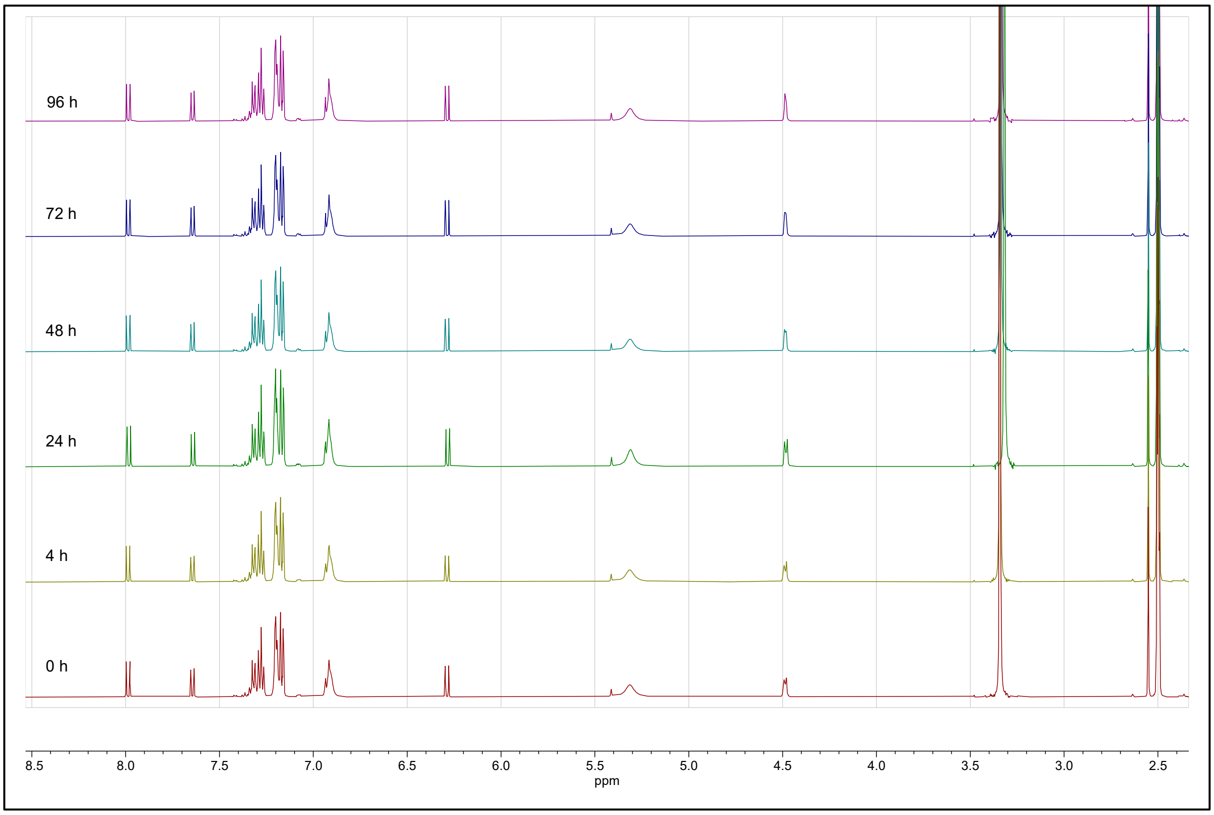


**Figure S3.6 a:** Stability of **11** examined in DMSO-$d_6$ (absence of light)

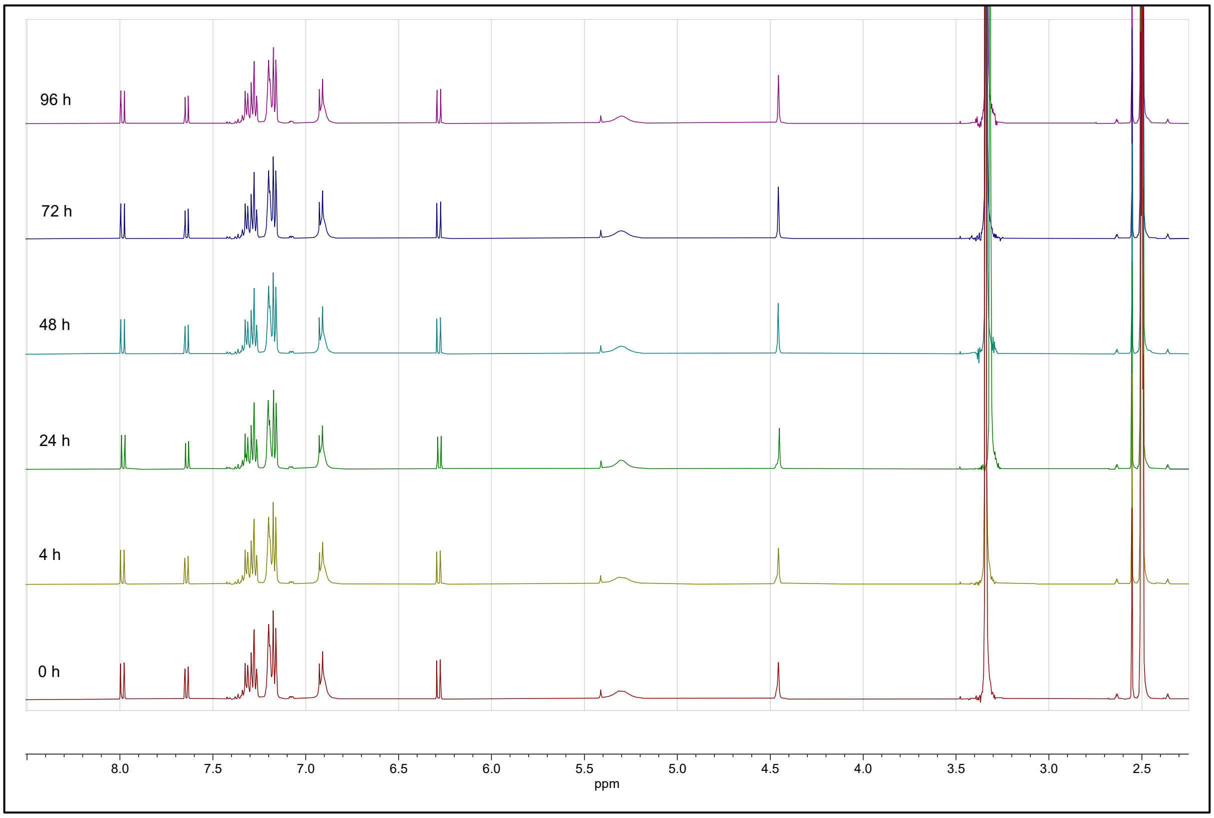


**Figure S3.6 b:** Stability of **11** examined in DMSO-$d_6$ (presence of UV/Vis light)

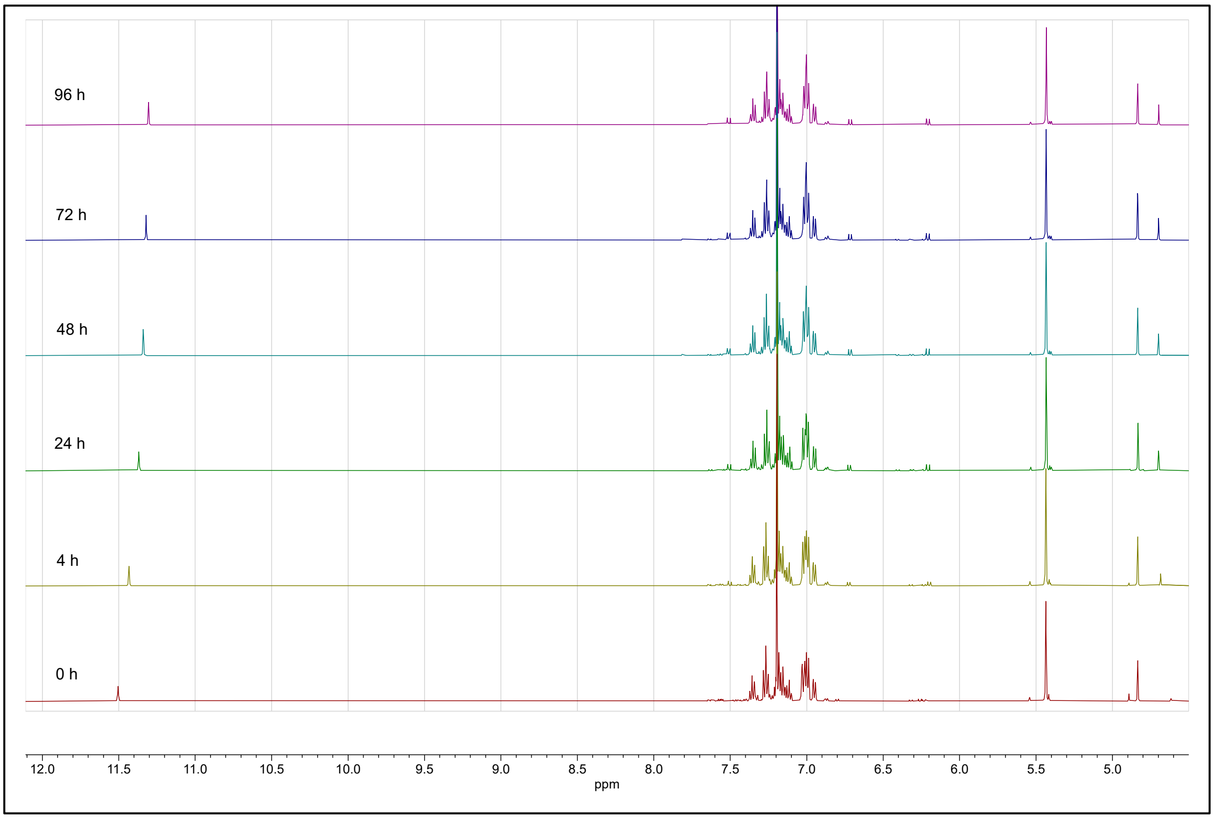


**Figure S3.6 c:** Stability of **11** examined in $CDCl_3$ (absence of light)

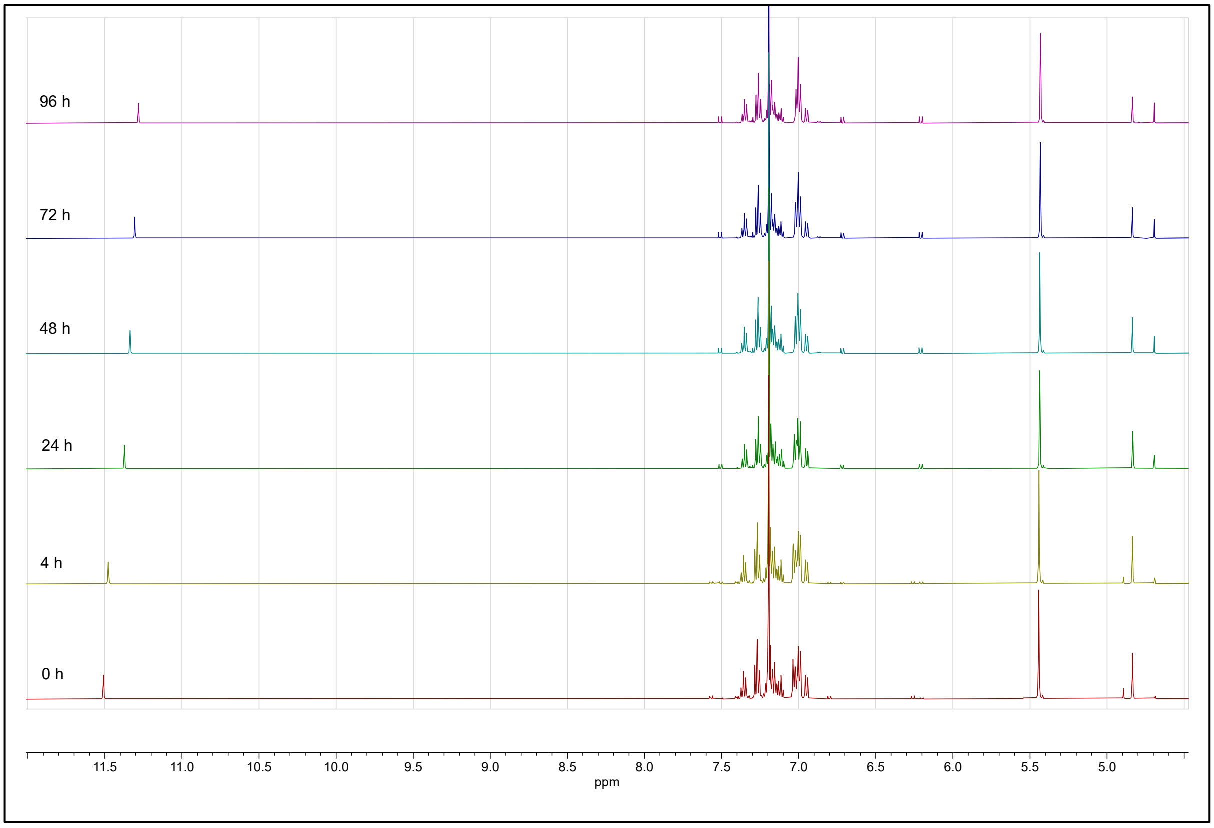


**Figure S3.6 d:** Stability of **11** examined in $CDCl_3$ (presence of UV/Vis light)

S4-Tables

**Table S4.1: Compound names and abbreviations**

| Compound name | Compound abbreviation |
|---|---|
| Ethyl-2-[8-acetyl-2-oxo-2H-chromen-7-yl)oxy]acetic acid | 8acetyl-C-7-oxyacetH **(1)** |
| Ethyl-2-[2-oxo-2H-chromen-7-yl)oxy]acetic acid | C-7-oxyacetH **(2)** |
| 2-(8-Acetyl-2-oxo-2H-chromen-7-yl)oxyacetosilver(I) | (8acetyl-C-7-oxyaceto)Ag **(3)** |
| 2-(2-Oxo-2H-chromen-7-yl)oxyacetosilver(I) | (C-7-oxyaceto)Ag **(4)** |
| [(bis-Triphenylphosphino)-(2-(8-Acetyl-2-oxo-2H-chromen-7-yl)[oxyacetosilver(I)} | [8acetyl-C-7-oxyacetoAgTPP$_2$] **(5)** |
| [(bis-Triphenylphosphino)-(2-(2-Oxo-2H-chromen-7-yl)oxyacetosilver(I)] | [C-7-oxyacetoAgTPP$_2$] **(6)** |
| 2-(8-Acetyl-2-oxo-2H-chromen-7-yl)oxyaceto sodium salt | [8-acetyl-C-7-oxyacetoNa] **(7)** |
| 2-(2-Oxo-2H-chromen-7-yl)oxyaceto sodium salt | [C-7-oxyacetoNa] **(8)** |
| [1,3-dibenzyl-4,5-diphenylimidazole-2-ylidene)]$^+$-[(8-acetylcoumarin-7-oxyacetate)$^-$ | [8-acetylcou-7-oxyacetNHC] **(9)** |
| [1,3-dibenzyl-4,5-diphenylimidazole-2-ylidene)]$^+$-[Coumarin-7-oxyacetate]$^-$ | [cou-7-oxyacetNHC] **(10)** |
| [(1,3-dibenzyl-4,5-diphenyl imidazole-2-ylidene)–(8-acetyl-2-oxo-2H-chromene-7-yl)]oxyaceto silver(I) | [(8-acetyl-C-7-oxyaceto)(Ag)(NHC)] **(11)** |
| [2-oxo-2H-chromen-3-carboxylato] silver(I) | [cou-3-Ag(I)] **(12)** |

**Table S4.2:** Experimental yields (%), melting points (°C) and main IR bands of compounds **1-4**

| Experimental Results | | | | | | |
|---|---|---|---|---|---|---|
| **Compound** | **Yield (%)** | **Melting point (°C)** | **Main I.R. bands ($cm^{-1}$)** | | | |
| | | | $\nu_{O-H}$ | $\nu_{C=O ester/lactone}$ | $\nu_{COO}$ (asym) | $\nu_{COO}$ (asym) |
| 8acetyl-C-7-oxyacetH **(1)** | 62 | 217.0 – 221.0 | 3071 | 1761 | | |
| C-7-oxyacetH **(2)** | 84 | 216.8 – 219.2 | 3078 | 1730 | | |
| (8acetyl-C-7-oxyaceto)Ag **(3)**[1] | 50 | 262.3 – 263.4 | - | 1719 | 1570 | 1405 |
| (C-7-oxyaceto)Ag **(4)** | 72 | 270.2 – 273.3 | - | 1727 | 1560 | 1406 |

**Table S4.3:** Experimental and literature values of **1-6**, showing chemical shifts (ppm), multiplicity patterns and *J* values (Hz); recorded in DMSO-$d_6$; OacetH gp. (oxyacetic acid group) = $-OCH_2COOH$; Acet. gp (acetyl group) = $-COOCH_3$

| **Compound** | **H³** | **H⁴** | **H⁵** | **H⁶** | **H⁷** | **H⁸** | **H¹¹** | **H¹²** | **H¹⁴** | **H¹⁶** | **H^TPP** |
|---|---|---|---|---|---|---|---|---|---|---|---|
| 8acetyl-C-7-oxyacetH **(1)** | 6.48, d, *J* = 9.5 | 8.02, d, *J* = 9.7 | 7.73, d, *J* = 8.6 | 7.09, d, *J* = 8.8 | OacetH. | Acet. gp. | 4.91, s | 13.24, s | 2.55, s | - | - |
| Literature values[7] | 6.35, d, *J* = 9.5 | 8.01, d, *J* = 9.7 | 7.73, d, *J* = 8.8 | 7.09, d, *J* = 8.8 | OacetH. | Acet. gp. | 4.91, s | N/R | 2.55, s | - | - |
| C-7-oxyacetH **(2)** | 6.30, d, *J* = 9.5 | 7.99, d *J* = 9.4 | 7.64, m, | 6.97-6.95, m | OacetH | 6.97-6.95, m | 4.83, s | 13.14, s | - | - | - |
| Literature values[7] | 6.28, d, *J* = 9.6 | 7.98, d, *J* = 9.6 | 7.62, d, *J* = 8.4 | 6.93, d, *J* = 8.4, | OacetH | 6.94, s | 4.81, s | N/R | - | - | - |
| (8acetyl-C-7-oxyaceto)Ag **(3)** | 6.30, d, *J* = 9.6 | 7.99, d, *J* = 8.8 | 7.66, d, *J* = 8.8 | 6.95, d, *J* = 9.5 | | Acet. gp. | 4.60, s | | | 2.57, s | |
| Literature values[7] | 6.28, d, *J* = 9.5 | 7.96, d, *J* = 9.6 | 7.64, d, *J* = 8.8 | 6.96, d, *J* = 8.8 | | Acet. gp. | 4.61, s | | | 2.56, s | |
| (C-7-oxyaceto)Ag **(4)** | 6.25, d, *J* = 9.5 | 7.97, d *J* = 9.4 | 7.58, d, *J* = 8.7 | 6.88, dd, *J* = 8.7, 2.4 | | 6.80, d, *J* = 2.5 | 4.54, s | | | | |
| Literature values[7] | 6.25, d, *J* = 9.5 | 7.96, d *J* = 9.4 | 7.58, d, *J* = 8.7 | 6.87, dd, *J* = 8.6, 2.4 | | 6.78, d, *J* = 2.4 | 4.51, s | | | | |
| [8acetyl-C-7-oxyacetoAgTPP$_2$] **(5)** | 6.27, d, *J* = 9.6 | 7.95, d, *J* = 9.6 | 7.42, d, *J* = 8.8 | 6.84, d, *J* = 8.8 | | Acet gp | 4.49, s | | | 2.54, s | 7.49-7.46 7.39-7.30 |
| [C-7-oxyacetoAgTPP$_2$] **(6)** | 6.24, d, *J* = 9.5 | 7.96, d, *J* = 9.5 | 7.53-7.35, m | 6.82, dd, *J* = 8.7, 2.5 | | 6.74, d, *J* = 2.4 | 4.42, s | | | - | 7.53-7.35, m |

N/R: not reported

**Table S4.4:** $^{13}$C NMR data of **1-6** showing the chemical shifts (ppm) recorded in DMSO-$d_6$

| Compound | $C^2$ | $C^3$ | $C^4$ | $C^5$ | $C^6$ | $C^7$ | $C^8$ | $C^9$ | $C^{10}$ | $C^{11}$ | $C^{12}$ | $C^{13}$ | $C^{14}$ | $C^{15}$ | $C^{16}$ | TPP CH ortho | TPP CH meta | TPP CH para | TPP QC |
|---|---|---|---|---|---|---|---|---|---|---|---|---|---|---|---|---|---|---|---|
| 8acetyl-C-7-oxyacetH **(1)** | 169.5 | 113.4 | 144.1 | 130.2 | 109.2 | 156.7 | 118.7 | 150.3 | 113.1 | 65.1 | 159.4 | 198.8 | 32.1 | - | - | - | - | - | - |
| C-7-oxyacetH **(2)** | 160.9 | 112.6 | 144.3 | 129.5 | 112.7 | 160.2 | 101.5 | 155.2 | 112.8 | 64.8 | 169.6 | - | - | - | - | - | - | - | - |
| (8acetyl-C-7-oxyaceto) Ag **(3)** | 159.6 | 112.7 | 144.4 | 129.9 | 109.7 | 158.3 | 118.5 | 150.2 | 112.2 | 68.0 | 170.0 | 190.4 | 32.2 | - | - | - | - | - | - |
| (C-7-oxyaceto)Ag **(4)** | 161.9 | 112.1 | 144.4 | 129.2 | 112.8 | 160.3 | 101.3 | 155.2 | 112.0 | 67.5 | 170.8 | - | - | - | - | - | - | - | - |
| [8acetyl-C-7-oxyaceto Ag(TPP$_2$)] **(5)** | 159.6 | 111.9 | 144.4 | 129.8 | 108.9 | 158.3 | 117.8 | 150.2 | 111.4 | 67.5 | 170.5 | - | - | 198.8 | 31.4 | 132.9-132.8 | 128.4-128.3 | 129.1 | 131.6-131.4 |
| [C-7-oxyacetoAg(TPP$_2$)] **(6)** | 162.3 | 111.9 | 144.4 | 130.6 | 112.9 | 160.4 | 101.2 | 155.3 | 111.8 | 67.9 | 170.7 | - | - | - | - | 133.6-133.5 | 129.1-129.0 | N/O | 131.9-131.7 |

N/O = not observed

**Table S4.5:** Crystallographic and structural refinement data for **5** and **6**

| **Name** | **5** | **6** |
|---|---|---|
| CCDC number | | |
| Empirical formula | $C_{49.37}$ $H_{39.99}$ $O_{6.12}$ $P_2$ Ag $Cl_{0.25}$ | $C_{46}$ $H_{37}$ $O_5$ $P_2$ Ag x $CH_2$ $Cl_2$ |
| Formula weight | 910.01 | 936.50 |
| Temperature [K] | 150(2) K | 100(2) K |
| Crystal system | Triclinic | Triclinic |
| Space group (number) | $P_1$ | $P_1$ |
| *a* [Å] | 13.0422(6) Å | 9.1382(4) Å |
| *b* [Å] | 13.3314(7) Å | 13.9517(7) Å |
| *c* [Å] | 14.5319(7) Å | 18.1986(9) Å |
| α [°] | 97.2432(17)° | 71.817(2)° |
| β [°] | 112.6229(15)° | 75.602(2)° |
| γ [°] | 107.4913(18)° | 72.632(2)° |
| Volume [$Å^3$] | 2138.95(18) $Å^3$ | 2072.67(18) $Å^3$ |
| *Z* | 2 | 2 |
| Density (calculated) | 1.413 $Mg/m^3$ | 1.501 $Mg/m^3$ |
| Absorption coefficient | 0.612 $mm^{-1}$ | 0.741 $mm^{-1}$ |
| F(000) | 933 | 956 |
| Crystal size | 0.163 x 0.116 x 0.06 $mm^3$ | 0.344 x 0.079 x 0.07 $mm^3$ |
| Theta range for data collection | 1.955 to 27.284° | 2.215 to 28.402° |
| Index ranges | –16<=h<=16, –17<=k<=17, –18<=l<=18 | –12<=h<=12, –18<=k<=18, –24<=l<=24 |
| Reflections collected | 37781 | 56106 |
| Independent reflections | 9585 [R(int) = 0.0685] | 10364 [R(int) = 0.0685] |
| Max. and Min. transmission | 0.7455and 0.7110 | 0.7457 and 0.6849 |
| Refinement method | Full–matrix least–squares on $F^2$ | Full–matrix least–squares on $F^2$ |
| Data / restraints / parameters | 9585 / 95 / 579 | 10364 / 0 / 524 |
| Goodness–of–fit on $F^2$ | 1.025 | 1.025 |
| Final R indices [I>2sigma(I)] | R1 = 0.0437, wR2 = 0.0798 | R1 = 0.0368, wR2 = 0.0593 |
| R indices (all data) | R1 = 0.0738, wR2 = 0.0904 | R1 = 0.0609, wR2 = 0.0669 |
| Extinction coefficient | 0.0014(3) | 0.00132(9) |
| Largest diff. peak and hole | 0.699 and –0.527 e.$Å^{-3}$ | 0.619 and –0.509 e.$Å^{-3}$ |

**Table S4.6:** NMR spectral data for compounds **9-11** showing the chemical shifts (ppm), multiplicity patterns and J-values (Hz); recorded in DMSO-$d_6$; *n/o = not observed

| | Compound | | | | | |
|---|---|---|---|---|---|---|
| | 9 | | 10 | | 11 | |
| Signal | H | C | H | C | H | C |
| 2 | - | 159.7 | - | 160.9 | - | 159.7 |
| 3 | 6.28, d, *J* = 9.5 | 112.31 | 6.24, d, *J* = 9.5 | 112.2 | 6.28, d, J = 9.5 | 112.6 |
| 4 | 7.99, d, *J* = 9.5 | 144.4 | 7.96, d, *J* = 9.9 | 144.9 | 7.98, d, J = 9.5 | 144.4 |
| 5 | 7.64, d, *J* = 8.7 | 130.2 | 7.57, d, *J* = 8.7 | 129.5 | 7.65, d, J = 8.7 | 130.6 |
| 6 | 6.90, d, *J* = 8.7 | 109.9 | 6.85, dd, *J* = 8.7, 2.4 | 113.3 | 6.96-6.90, m | 109.7 |
| 7 | - | 158.8 | - | 163.1 | - | 158.3 |
| 8 | - | 118.3 | 6.76, d, J = 2.5 | 101.7 | - | 118.4 |
| 9 | - | 150.2 | - | 155.7 | - | 150.2 |
| 10 | - | 111.8 | - | 112.1 | - | 112.14 |
| 11 | 4.37, s | 68.3 | 4.38, s | 68.6 | 4.54, s | 67.9 |
| 12 | - | 168.3 | - | 169.1 | - | 170.6 |
| 13 | - | 199.6 | - | - | - | 199.4 |
| 14 | 2.57, s | 32.1 | - | - | 2.55,s | 32.1 |
| 15 | 9.72, s | 136.7 | 9.73, s | 137.3 | - | 136.7 |
| 16 | - | 134.1 | - | 134.5 | - | 132.2 |
| 17 | 5.42, s | 50.5 | 5.42, s | 50.9 | 5.33, s | 52.6 |
| 18 | - | 124.9 | - | 125.4 | - | 127.5 |
| 19 | 7.46-7.26, m | 130.8 | 7.45-7.26, m | 131.3 | 7.35-7.15, m | 127.7 |
| 20 | | 129.8 | | 130.6 | 7.35-7.15, m | 128.5 |
| 21 | n/o | 127.8 | n/o | 129.3 | 6.96-6.90, m | 126.8 |
| 22 | - | 131.8 | - | 132.3 | - | 128.8 |
| 23 | 7.46-7.26, m | 128.5 | 7.45-7.26, m | 128.3 | 7.35-7.15, m | 129.9 |
| 24 | 7.08, m | 128.8 | 7.12-7.04, m | 128.9 | 7.35-7.15, m | 129.2 |
| 25 | | | | | 6.96-6.90, m | 126.8 |

Table S4.7: **Table of MS Data for Adduct 11**

| **8-acetylcou-7-oxyacetoAgNHC** | | | | |
|---|---|---|---|---|
| **Formula** | **predicted (m/z)** | **found(m/z)** | **diff (ppm)** | |
| C13H106 | 263.0555 | 263.0565 | 3.801479156 | 8-acetylcou-7-oxyacetoH |
| C29H24N2 | 401.2012 | 401.2038 | 6.480538942 | NHC |
| C29H24N2Ag | 507.099 | 507.1008 | 3.54960274 | NHC-Ag+ |
| C58H48N4Ag | 907.293 | 907.2964 | 3.747411255 | [NHC-Ag-NHC]+ |

Table S4.8 Growth Curves for ligand **2** against *E. coli*, MRSA and *P. aeruginosa*, measured using the Micro Broth Dilution Assay.

# *E. coli*

| | Coumarin-7-oxyacetic acid | | | | | | | | | | | |
|---|---|---|---|---|---|---|---|---|---|---|---|---|
| Concentration(ug/mL) | 0 | 1 | 2 | 4 | 8 | 16 | 32 | 64 | 128 | 256 | 512 | 1024 |
| n1 | 1.656 | 1.812 | 1.712 | 1.957 | 1.892 | 1.799 | 1.795 | 1.935 | 1.853 | 1.933 | 1.716 | 0.98 |
| | 1.694 | 1.621 | 1.604 | 1.7 | 1.893 | 1.931 | 1.968 | 1.823 | 1.971 | 1.994 | 1.819 | 0.929 |
| n2 | 1.285 | 1.004 | 0.971 | 1.252 | 1.324 | 1.433 | 1.411 | 1.485 | 1.233 | 1.566 | 1.6 | 1.438 |
| | 1.361 | 0.794 | 0.902 | 0.121 | 0.108 | 0.099 | 0.102 | 0.109 | 0.097 | 0.097 | 0.104 | 0.583 |
| n3 | 1.525 | 1.311 | 1.332 | 1.344 | 1.357 | 1.405 | 1.397 | 1.71 | 1.654 | 1.746 | 1.809 | 1.495 |
| | 1.374 | 1.353 | 1.302 | 1.359 | 1.449 | 1.409 | 1.486 | 1.524 | 1.493 | 1.69 | 1.679 | 1.339 |
| Average OD | 1.4825 | 1.315833 | 1.303833 | 1.288833 | 1.337167 | 1.346 | 1.359833 | 1.431 | 1.3835 | 1.504333 | 1.4545 | 1.127333 |
| St Dev | 0.168631 | 0.376899 | 0.325498 | 0.63066 | 0.654326 | 0.650763 | 0.657465 | 0.67004 | 0.682419 | 0.707219 | 0.666683 | 0.356044 |
| %Growth | 100 | 88.75773 | 99.08803 | 86.93648 | 90.19674 | 90.79258 | 91.72569 | 96.52614 | 93.32209 | 101.4727 | 98.1113 | 76.04272 |

# MRSA

| | Coumarin-7-oxyacetic acid | | | | | | | | | | | |
|---|---|---|---|---|---|---|---|---|---|---|---|---|
| Concentration(ug/mL) | 0 | 1 | 2 | 4 | 8 | 16 | 32 | 64 | 128 | 256 | 512 | 1024 |
| n1 | 0.765 | 0.791 | 0.849 | 0.766 | 0.838 | 0.892 | 0.744 | 0.665 | 0.803 | 0.681 | 0.806 | 0.617 |
| | 0.644 | 0.872 | 0.883 | 0.848 | 0.842 | 0.763 | 0.75 | 0.606 | 0.79 | 0.798 | 0.761 | 0.629 |
| n2 | 0.546 | 0.541 | 0.578 | 0.706 | 0.728 | 0.887 | 0.711 | 0.475 | 0.417 | 0.783 | 0.505 | 0.482 |
| | 0.634 | 0.665 | 0.779 | 0.61 | 0.733 | 0.944 | 0.792 | 0.627 | 0.801 | 0.806 | 0.684 | 0.31 |
| n3 | 0.62 | 0.739 | 0.807 | 0.675 | 0.918 | 0.752 | 0.736 | 0.634 | 0.705 | 0.842 | 0.926 | 0.719 |
| | 0.409 | 0.779 | 0.912 | 0.743 | 0.752 | 0.647 | 0.63 | 0.803 | 0.797 | 0.823 | 0.828 | 0.74 |
| Average OD | 0.603 | 0.731167 | 0.801333 | 0.724667 | 0.801833 | 0.814167 | 0.727167 | 0.635 | 0.718833 | 0.788833 | 0.751667 | 0.582833 |
| St Dev | 0.118372 | 0.115156 | 0.119664 | 0.08151 | 0.076274 | 0.111935 | 0.054385 | 0.10548 | 0.152515 | 0.05663 | 0.144694 | 0.161955 |
| %Growth | 100 | 121.2548 | 109.5965 | 120.1769 | 132.974 | 135.0193 | 120.5915 | 105.3068 | 119.2095 | 130.8181 | 124.6545 | 96.65561 |

# *P. aeruginosa*

| | Coumarin-7-oxyacetic acid | | | | | | | | | | | |
|---|---|---|---|---|---|---|---|---|---|---|---|---|
| Concentration(ug/mL) | 0 | 1 | 2 | 4 | 8 | 16 | 32 | 64 | 128 | 256 | 512 | 1024 |
| n1 | 1.069 | 1.176 | 1.247 | 1.26 | 1.359 | 1.293 | 1.353 | 1.216 | 1.097 | 1.157 | 0.961 | 0.816 |
| | 1.184 | 1.149 | 1.338 | 1.309 | 1.377 | 1.381 | 1.487 | 1.295 | 1.195 | 1.315 | 1.315 | 0.885 |
| n2 | 1.449 | 1.293 | 1.271 | 1.235 | 1.321 | 1.314 | 1.33 | 1.366 | 1.37 | 1.352 | 1.34 | 1.044 |
| | 1.515 | 1.246 | 1.261 | 1.214 | 1.31 | 1.273 | 1.38 | 1.434 | 1.462 | 1.355 | 1.372 | 1.036 |
| n3 | 0.915 | 1.033 | 1.134 | 1.218 | 1.189 | 1.096 | 1.239 | 1.204 | 1.075 | 1.14 | 0.928 | 0.784 |
| | 1.005 | 1.082 | 1.198 | 1.142 | 1.175 | 1.121 | 1.156 | 1.021 | 1.225 | 1.223 | 0.839 | 0.676 |
| Average OD | 1.1895 | 1.163167 | 1.2415 | 1.229667 | 1.2885 | 1.246333 | 1.324167 | 1.256 | 1.237333 | 1.257 | 1.125833 | 0.8735 |
| St Dev | 0.243816 | 0.097586 | 0.069342 | 0.055334 | 0.086143 | 0.113059 | 0.114918 | 0.144799 | 0.152358 | 0.096786 | 0.241177 | 0.145555 |
| %Growth | 100 | 97.78618 | 106.7345 | 103.3768 | 108.3228 | 104.7779 | 111.3213 | 105.5906 | 104.0213 | 105.6747 | 94.64761 | 73.43422 |
